# Global Gene Expression of Cultured Human Dermal Fibroblasts: Focus on Cell Cycle and Proliferation Status in Improving the Condition of Face Skin

**DOI:** 10.7150/ijms.46265

**Published:** 2021-02-03

**Authors:** Bogusław Machaliński, Dorota Rogińska, Aleksandra Wilk, Kamila Szumilas, Piotr Prowans, Edyta Paczkowska, Paweł Szumilas, Iwona Stecewicz, Piotr Zawodny, Maciej Ziętek, Barbara Wiszniewska

**Affiliations:** 1 Department of General Pathology, Pomeranian Medical University, Powstanców Wlkp. 72, 70-111 Szczecin, Poland.; 2 Department of Histology and Embryology, Pomeranian Medical University, Powstanców Wlkp. 72, 70-111 Szczecin, Poland.; 3 Department of Physiology, Pomeranian Medical University, Powstanców Wlkp. 72, 70-111 Szczecin, Poland.; 4 Department of Social Medicine and Public Health, Chair of Social Medicine, Pomeranian Medical University, Żołnierska 48, 71-210 Szczecin, Poland.

**Keywords:** autologous fibroblasts, transcriptome profile, qRT-PCR, cell cycle genes, proliferation status, reimplantation

## Abstract

Chronological skin ageing is an inevitable physiological process that results in thin and sagging skin, fine wrinkles, and gradual dermal atrophy. The main therapeutic approaches to soft tissue augmentation involve using dermal fillers, where natural fillers, such as autologous fibroblasts, are involved in generating dermal matrix proteins. The aim of this study was to determine the global transcriptome profile of three passages of dermal autologous fibroblasts from a male volunteer, focusing on the processes of the cell cycle and cell proliferation status to estimate the optimal passage of the tested cells with respect to their reimplantation.

We performed K-means clustering and validation of the expression of the selected mRNA by qRT-PCR. Ten genes were selected (ANLN, BUB1, CDC20, CCNA2, DLGAP5, MKI67, PLK1, PRC1, SPAG5, and TPX2) from the top five processes annotated to cluster 5. Detailed microarray analysis of the fibroblast genes indicated that the cell population of the third passage exhibited the highest number of upregulated genes involved in the cell cycle and cell proliferation. In all cases, the results of qRT-PCR confirmed the differences in expression of the selected mRNAs between fibroblasts from the primary culture (C0) and from the first (C1), second (C2), and third (C3) cell passage. Our results thus suggest that these cells might be useful for increasing fibroblast numbers after reimplantation into a recipient's skin, and the method used in this study seems to be an excellent tool for autologous transplantation allowing the rejuvenation of aging skin.

## Introduction

The intrinsic and extrinsic ageing of skin are associated with the most obvious changes in the dermis 1. The morphological changes seen in aged skin include a decrease in the number of fibroblasts compared to younger tissue. Moreover, there are quantitative and structural changes in collagen, elastic fibers, glycosaminoglycans, and proteoglycans of the extracellular matrix 2, 3. Chronological skin ageing is an inevitable physiological process that results in thin and sagging skin, fine wrinkles, and gradual dermal atrophy 4. Improving the appearance of skin for better psychosocial functioning—including such aspects as improved quality of life, self-esteem, and body image—is becoming more and more popular for both women and men 5. Surgical and minimally invasive facial cosmetic procedures for wrinkle correction and soft tissue augmentation have been used for a long time 5, 6.

Various antiaging approaches have been developed with the stated aim of achieving young and healthy skin 7. There are two types of antiaging approach that are widely used at present. The first involves application of the retinoid vitamin A, which increases the amount of type I collagen, stimulates elastic fiber organization and glycosaminoglycan synthesis, and decreases the amount of metalloproteinases 7, 8. The other approach uses topical cosmeceuticals containing ascorbic acid (vitamin C) as a cofactor in the hydroxylation process of procollagen, and antioxidants to enhance the resistance of skin to oxidative stress 9; topical α-hydroxy acids (AHA) are also used in the treatment of photoaging skin 10, and glycolic or lactic acids can stimulate glycosaminoglycans and collagen synthesis 7, 11. Energy-based dermal rejuvenation is a nonsurgical technique that uses various types of lasers to heat the dermis and stimulate matrix remodeling, inducing biosynthesis of type I and III collagens 7. Non-laser-based approaches include the radiofrequency (RF) method, referred to as a non-surgical facelift, and high-intensity focused ultrasound (HIFU). Both these methods provide significant improvements in skin firmness and laxity by stimulating type I and type III collagen synthesis and improving the quality of elastic fibers 7, 12.

Injection of dermal fillers is increasingly used in rejuvenating the face and hands, and is regarded as an extracellular microenvironment modulator 7. After the injection, these materials can fill rhytides, folds, and wrinkles, as well as replace the soft tissue that is gradually lost during chronological ageing 13. There are several fillers that not only provide soft tissue augmentation, but also stimulate collagen synthesis 14. The biodegradable fillers used for the rejuvenation of skin include collagen, hyaluronic acid, poly-L-lactic acid 15, and autologous fat fillers 16.

Because fibroblasts make up the main functional population of cells in the dermis, tissue engineering technology using autologous cultured fibroblasts has been developed, as more beneficial in facial rejuvenation than other therapies 10, 17. The method with autologous cultured fibroblasts has been used effectively for dermal and subcutaneous deficiencies since 1995 10, 18, 19. The ideal filler material would be nonallergenic, noncarcinogenic, and nonteratogenic 14. Autologous fibroblast therapy for the treatment of various wrinkles, depressed scars, and acne irregularities is considered to be safe and well tolerated, as has been confirmed in various studies 20-23.

The detailed transcriptome profile analysis presented in our previous study 24 indicated that the second passage of fibroblasts is the optimal population due to the secretion and organization of extracellular matrix elements for potential autologous reimplantation to the skin. The present study continues this research with the comprehensive aim of examining the autologous fibroblast transcriptome profile by focusing on the processes of the cell cycle and cell proliferation status, in order to estimate the optimal passage of the tested cells with respect to reimplantation.

## Material and Methods

The results presented in this paper continue on the gene expression profile analysis described by Machaliński et al. 24.

Briefly, four RNA samples were extracted from cultured dermal fibroblasts derived from a 46-year-old male patient at different time points—from the primary culture (C0), and from the first (C1), second (C2), and third (C3) passage of cells—and were used for whole transcriptome microarray analysis on the Affymetrix platform (Thermo Fisher Scientific, Waltham, MA, USA). A detailed description of these procedures, including cell culture conditions, total RNA isolation, and the determination of the complete list of differentially expressed genes (DEGs) can be found in the abovementioned publication.

### K-means clustering

To identify the subgroups of coregulated DEGs that behave similarly in all analyzed samples, we used the K-means clustering approach 25. Genes were defined as differentially expressed if the fold change value was higher than |2| in at least one of the comparison groups: C1 vs. C0, C2 vs. C0, or C3 vs. C0. First, the sum of the squared error (SSE) estimation was used to determine the optimal number of clusters. The expression value of DEGs was then scaled and centered to fit into the range <-1.5; 1.5> and subjected to the K-means clustering procedure. Subsequently, the correlation of each gene from the clusters with cluster centroid (cluster core) values was calculated. Genes with correlation scores below 0.8 were filtered out. The results were visualized as a heatmap and line graphs using the “pheatmap” and “ggplot2” libraries. Genes belonging to individual clusters were subjected to functional annotation analysis using the Database for Annotation, Visualization, and Integrated Discovery (DAVID) bioinformatics tool 26. Gene symbols from each cluster were uploaded to DAVID by the “RDAVIDWebService” BioConductor library 27, where they were assigned to corresponding gene ontology (GO) terms from the GO BP Direct database. We used the clusterProfiler tool to analyze and visualize the functional profile of gene clusters 28.

### Validation of expression of selected mRNA

qRT-PCR was used to evaluate the gene expression results obtained by microarray analysis. We selected the genes from the top five processes annotated to cluster 5: ANLN, BUB1, CDC20, CCNA2, DLGAP5, MKI67, PLK1, PRC1, SPAG5, and TPX2.

The primers for qRT-PCR were designed using Primer3web version 4.1.0 software 29-31 and were purchased from the Laboratory of DNA Sequencing and Oligonucleotide Synthesis, Institute of Biochemistry and Biophysics, Polish Academy of Sciences in Warsaw, Poland. The complete primers sequences, together with the annealing temperatures used in the qRT-PCR protocol, are listed in Table 1.

For quantitative assessment of selected gene expressions levels, the total RNA (100 ng) was reverse transcribed using a First Strand cDNA Synthesis Kit (Thermo Fisher Scientific, Waltham, MA, USA) and the cDNA thus obtained was used as a template for the subsequent reaction. The qRT-PCR was carried out using a CFX96 Touch Real-Time PCR Detection System (Bio-Rad, Hercules, CA, USA) and the reaction mixture consisted of 5 μL SYBR Green PCR Master Mix, 1 μL cDNA, 3.6 μL nuclease-free water; and 0.4 μL specific primers (0.2 μL forward primer and 0.2 μL reverse primer). The protocol began with five minutes of initial denaturation, followed by a three-step amplification program consisting of: denaturation at 95 °C for 15 s, annealing for 30 s at 59.2 °C, 60.1 °C or 62.0 °C (depending on the primer used), and extension at 60 °C for one minute. The specificity of the reaction products was checked by determining the melting points (0.1 °C/s transition rate). Relative gene expression was calculated using CFX Maestro software (Bio-Rad, Hercules, CA, USA) and the comparative Ct method (2ΔCt, where ΔCt = [Ct of target genes] - [Ct of endogenous control gene]).

## Results

### K-means clustering

**Figure 1:**
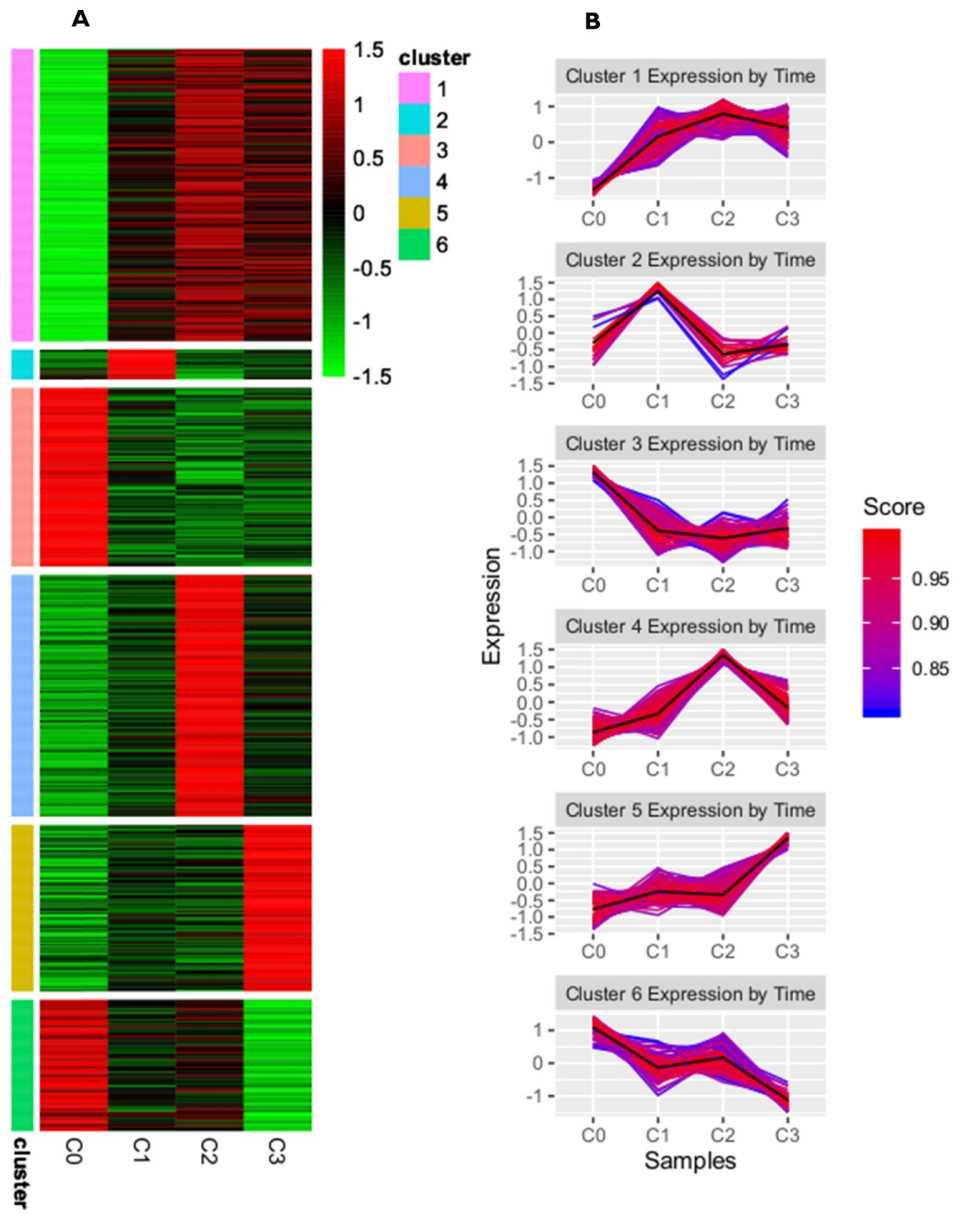
K-means clustering analysis of differentially expressed genes from skin fibroblasts obtained from primary culture (C0) and from the 1^st^ (C1), 2^nd^ (C2), and 3^rd^ (C3) cell passages.** (A)** Heatmap presenting up- and downregulated genes within six clusters for individual experimental samples. Each horizontal line represents a single DEG, and the color reflects the status of the genes in case of their up- (red color) and downregulation (green color); **(B)** Line graphs showing kinetics of changes in gene expression profile within a given cluster. The score value indicates the correlation of each gene from the clusters with the cluster centroid.

A complete list of differentially expressed genes (DEGs), both upregulated and downregulated, in the cultured dermal fibroblasts from the male volunteer is presented in the supplementary materials of our previously published manuscript 24. There, the DEGs of fibroblasts from the fist (C1), second (C2), and third (C3) cell passages were compared with those from the primary culture (C0). To identify the subgroups of coregulated DEGs that behave similarly in all the samples, we used a K-means clustering approach. On the basis of the sum of squared errors (SSE), the DEGs were divided into six clusters: cluster 1 included 167 genes, cluster 2 had 17 genes, cluster 3 had 102 genes, cluster 4 had 138 genes, cluster 5 had 95, and cluster 6 had 75 genes (see supplementary files). A heatmap representing upregulated and downregulated genes within all clusters, as well as the kinetics of gene expression changes in the primary culture and the subsequent passages of dermal fibroblasts, is shown in Figure 1A and B, respectively.

### DEGSs analysis in aspect of cell cycle and proliferation

**Figure 2:**
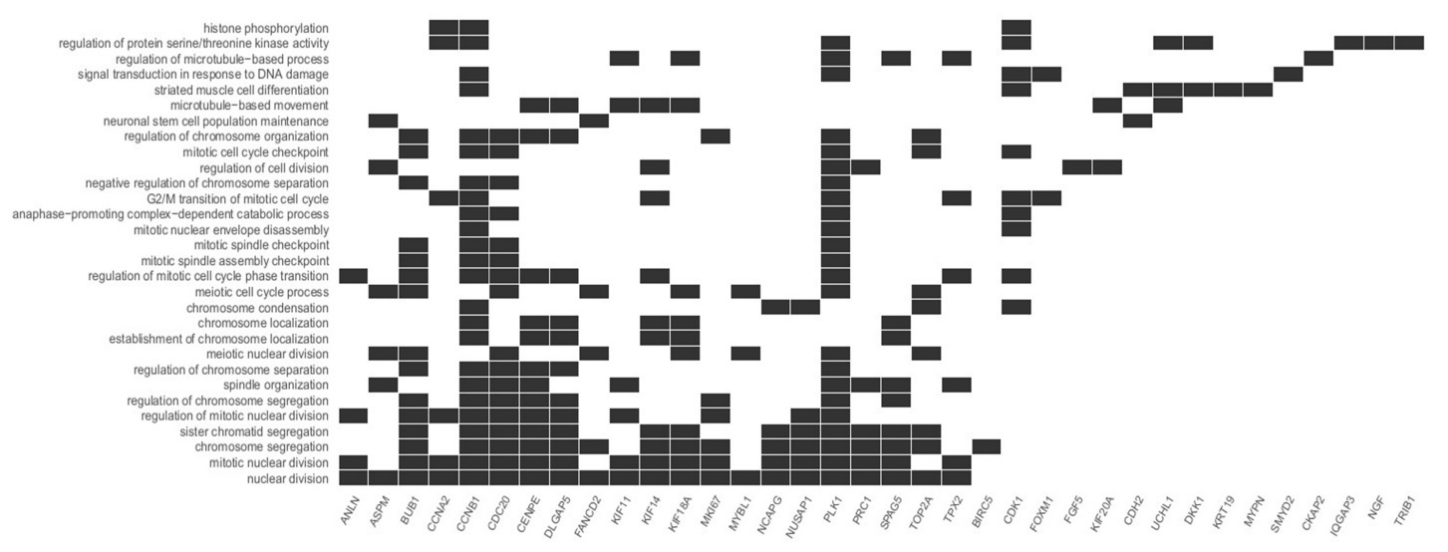
Diagram presenting processes included in cluster 5. Each black square represents a single gene annotated to a given biological process.

This study focuses on the analysis of DEGs involved in the cell cycle and proliferation, which were annotated in cluster 5. The highest rate of differentially expressed genes associated with these processes was observed in fibroblasts from the third passage (Figure 1A). Cluster analysis showed that the expression of genes was greater in the C3 passage of fibroblasts than in C0, C1, and C2 (Figure 1B). All the processes from cluster 5, together with corresponding DEGs, are presented in Figure 2.

### Top five processes involved in the cell cycle and proliferation

We focused on the five processes with the highest rates of upregulated genes involved in the cell cycle and proliferation: *nuclear division, mitotic nuclear division, chromosome segregation, sister chromatid segregation,* and* regulation of mitotic nuclear division.* The most represented was *the nuclear division* process, with 21 upregulated genes. The differentially expressed genes annotated to these processes are listed in Table 2.

**Figure 3:**
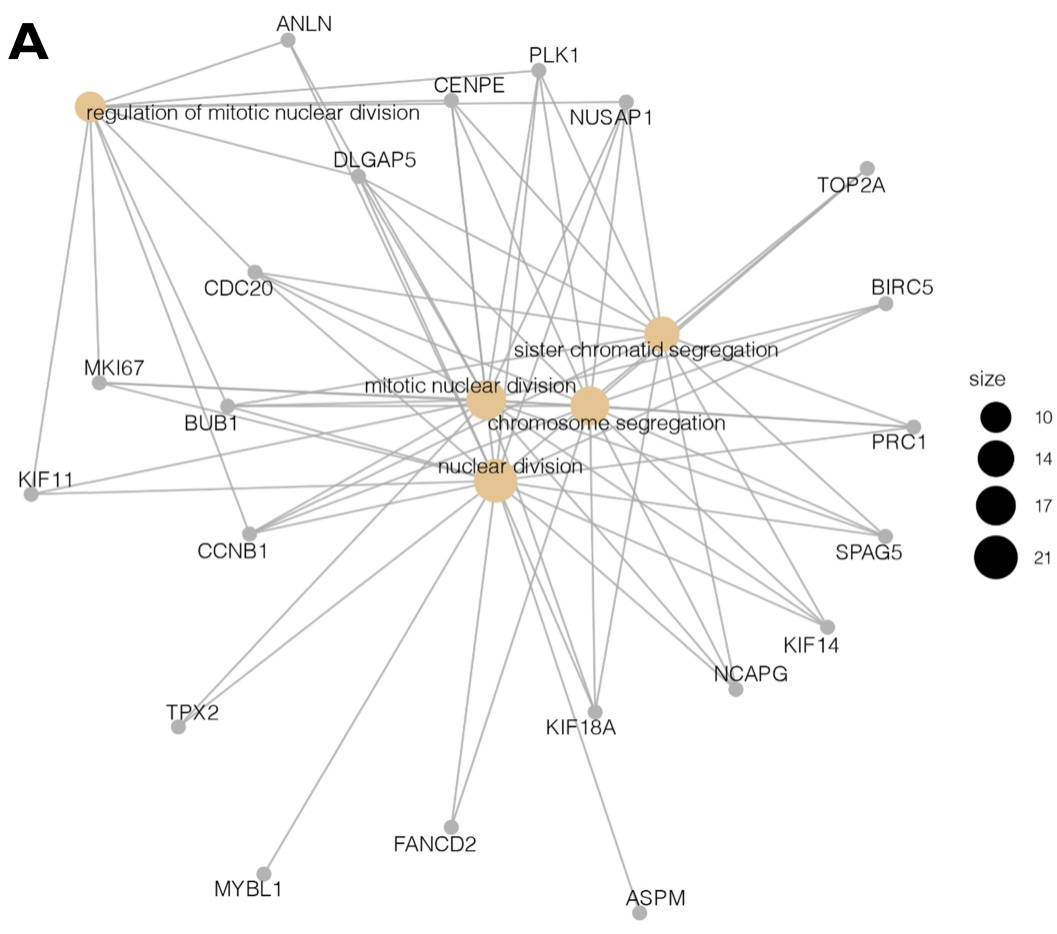

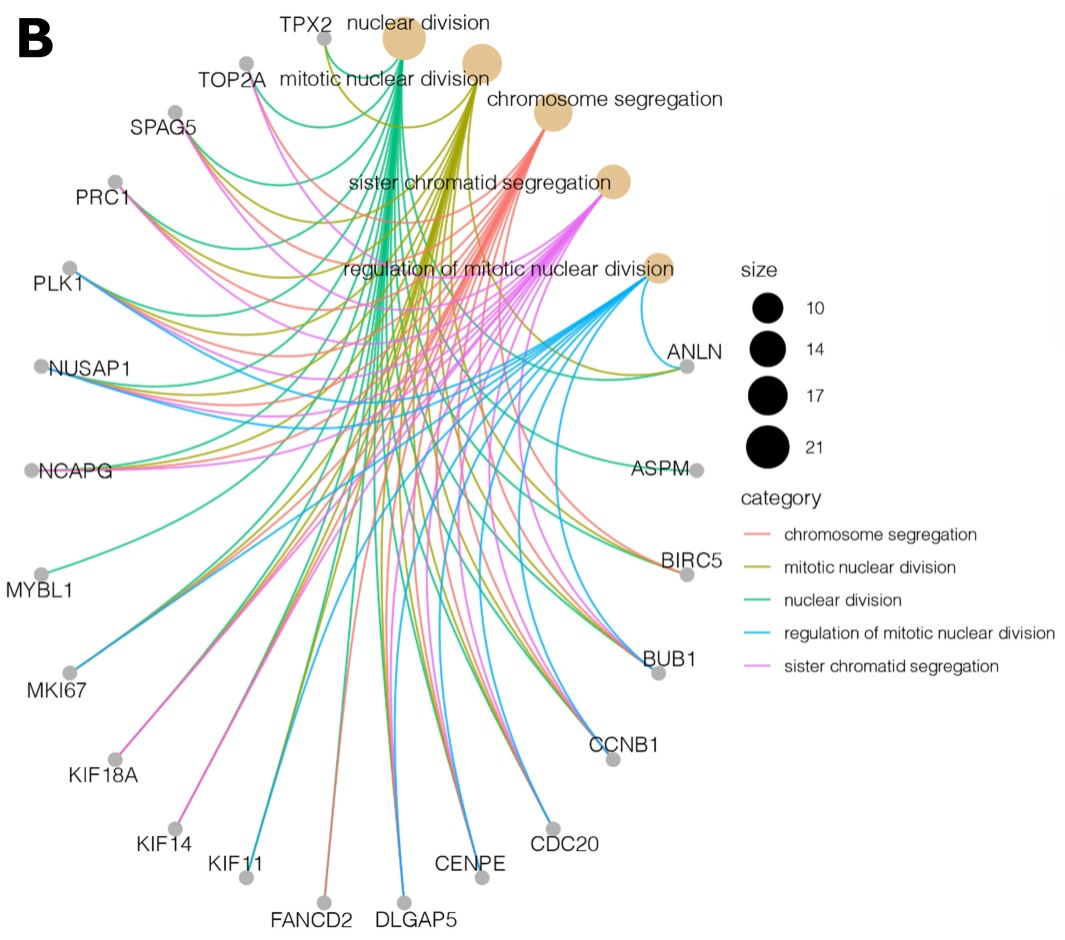
** (A, B)** Cluster profile analysis of the top 5 processes and the corresponding DEGs from cluster 5. The size of the circle indicates the number of genes represented in a given biological process.

Moreover, due to the ambiguous nature of the Gene Ontology database, some genes are assigned to more than one functional annotation: for example, the ***BUB1, CCNB1, CDC20, CENPE, DLGAP5, NUSAP1****,* and***PLK1***genes were mapped to the top five processes (Table 2). These processes with the involved genes are additionally shown in Figure 3 A, B.

### Comparative study of gene expression

**Figure 4:**
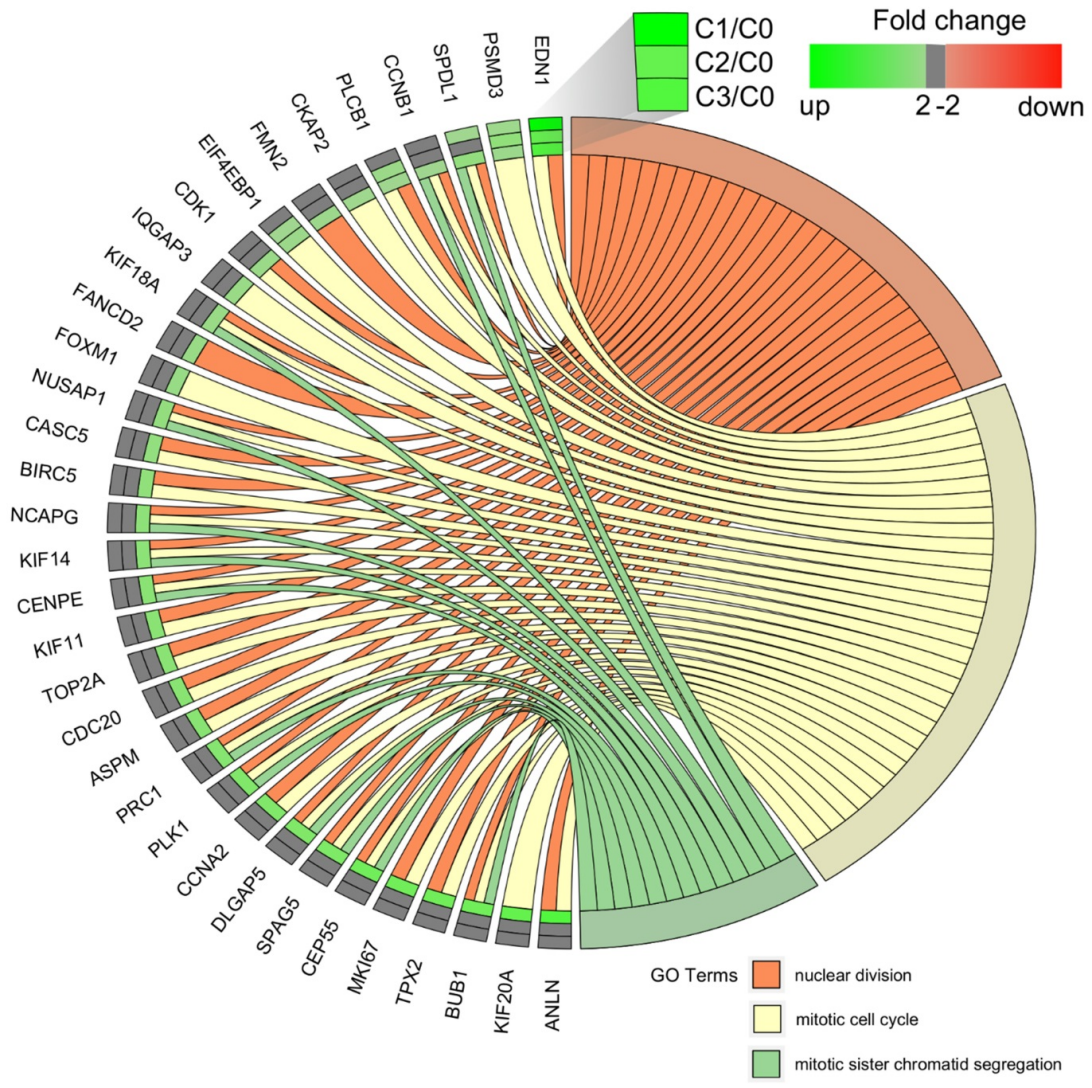
Circular genome data visualization (circos) plot for the selected overrepresented Gene Ontology (GO) terms and corresponding differentially expressed genes (DEGs). The relevant fold change values are presented by the color scale (green - upregulated, red - downregulated, grey - unchanged), where external rectangles indicate C1 vs. C0, inner rectangles indicate C2 vs. C0 and internal rectangles indicate C3 vs. C0 comparison groups.

We carried out a complementary comparative study of the gene expression profile in order verify that the analysis had been performed correctly (Figure 4). DAVID was used to identify functional annotations of DEGs from the three passages, compared with those in the primary culture. We focused on the three processes directly associated with the cell cycle and proliferation: *nuclear division, mitotic cell cycle*, and* mitotic sister chromatid segregation*. In comparing the three passages by primary culture, C3/C0 showed the greatest accumulation of upregulated genes associated with the cell cycle and proliferation, which is shown in circular genome data visualization (circos plot) (Figure 4).

The diagrams show that the greatest accumulation of upregulated cell cycle-related genes in fibroblasts of the third passage are grouped and that, according to the Gene Ontology classification, these 22 genes are involved in the following five processes: (i) nuclear division (21 genes), (ii) mitotic nuclear division (17 genes), (iii) chromosome segregation (16 genes), (iv) sister chromatid segregation (13 genes), and (v) regulation of mitotic nuclear division (11 genes). Interestingly, the following genes were common to all five processes: *BUB1, CCNB1, CDC20, CENPE, DLGAP5, NUSAP1,* and *PLK1*. The characterization of the 22 upregulated genes is presented in Table 3.

### Assessment of cellular senescence

**Figure 5:**
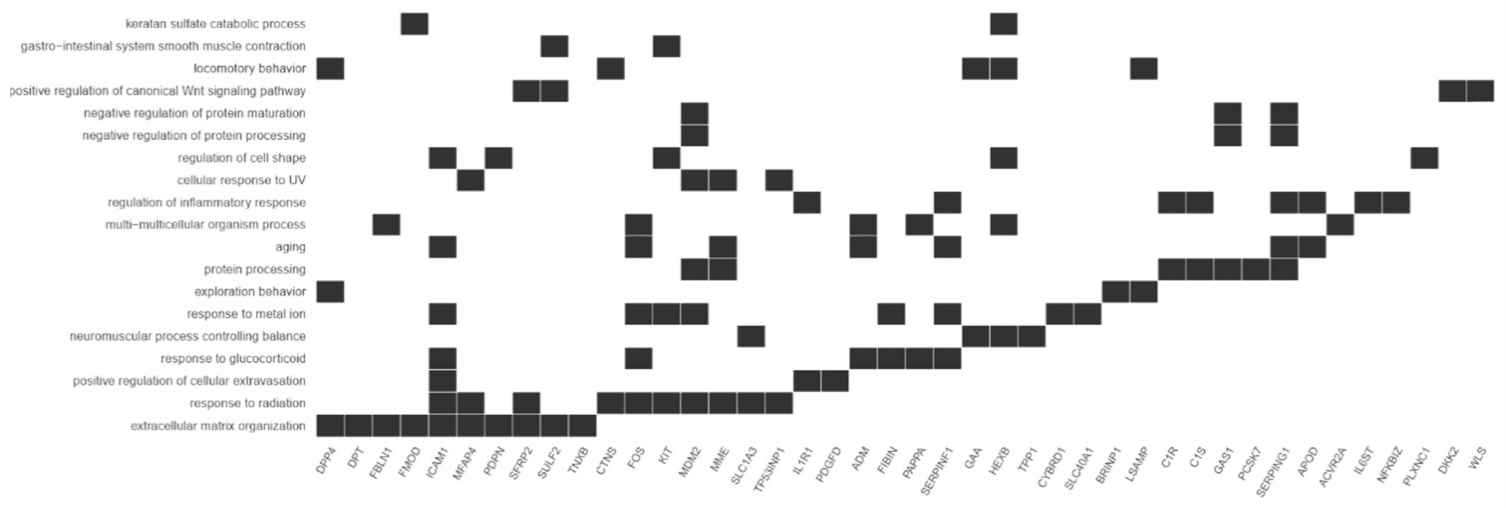
Diagram presenting processes included in cluster 6. Each black square represents a single gene annotated to a given biological process.

When reimplementing fibroblasts for skin rejuvenation, it is important to use cells that are viable and show no signs of cellular aging. In our study, we found a couple of genes associated with aging process, which were assigned to cluster 6: *ADM* (adrenomedullin), *APOD* (apolipoprotein D), *FOS* (FBJ murine osteosarcoma viral oncogene homolog B), *ICAM1* (intercellular adhesion molecule 1), *MME* (membrane metallo-endopeptidase), *SERPINF1* (serpin peptidase inhibitor, clade F (alpha-2 antiplasmin, pigment epithelium derived factor), member 1) and *SERPING 1* (serpin peptidase inhibitor, clade G (C1 inhibitor), member 1). The expression of this genes was either downregulated or unchanged during all tested cell passages, however the lowest expression was observed in the fibroblasts from 3^rd^ passage (see supplementary files). All the processes from cluster 6, including aging are presented in Figure 5.

It is worth noting that the presented results are derived from cells at an early stage of culture and the expression of genes related to cellular aging would probably be increased in later passages.

### Verification of microarray results using qRT-PCR

**Figure 6:**
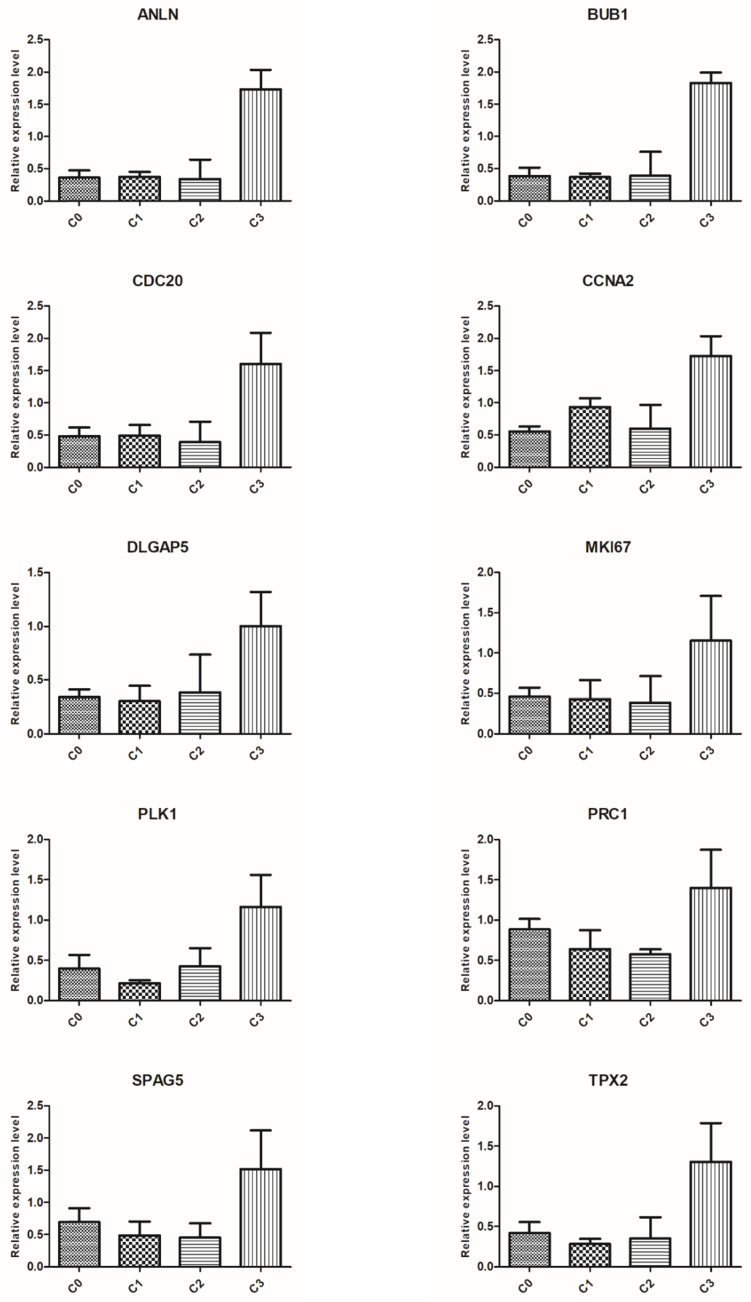
Real-time quantitation of selected genes: ANLN, BUB1, CDC20, CCNA2, DLGAP5, MKI67, PLK1, PRC1, SPAG5 and TPX2 in human dermal fibroblast from primary culture (C0) and the 1st (C1), 2nd (C2), and 3rd (C3) cell passage. The expression level of all examined genes was the highest in the cells from the third passage, which confirms the results revealed by microarray assay. Data are presented as the mean ± SD (n = 3). Charts were generated using GraphPad Prism software.

To validate the results of microarray analysis, we selected ten genes from the top five processes annotated to cluster 5: ANLN, BUB1, CDC20, CCNA2, DLGAP5, MKI67, PLK1, PRC1, SPAG5, and TPX2, and performed qRT-PCR on them. In all cases, qRT-PCR confirmed the differences in expression of selected mRNAs between fibroblasts from primary culture (C0) and those from the first (C1), second (C2), and third (C3) cell passages, as revealed by the global gene expression analysis (Figure 6).

## Discussion

Mitotic cell division is a common process in human somatic cells that includes a series of events called the cell cycle. The cell cycle is regarded as the most fundamental of all biological processes and can be classified into four sequential phases: G1 (first gap phase), S (DNA replication), G2 (second gap phase), and M (mitosis). The gap phases (G1 and G2) are not periods of inactivity: rather, during these phases, cells gain mass, integrate growth signals, organize the replicated genome, and prepare for chromosome segregation 32. This process requires numerous genes that are transcriptionally regulated and associated with this phase of the cell cycle 33. Cells with the ability to renew themselves are used in cell culture *ex vivo* for successful cell therapy, not only in reconstructive or regenerative medicine, but also in aesthetic medicine 34.

Fibroblasts are the main functional cells in the dermis, and injection of autologous fibroblasts is regarded to be more beneficial and more persistent than other therapies, such as the various dermal fillers. This type of therapy has been effectively used for dermal and subcutaneous deficiencies since 1995 18, 35, and clinical research into for autologous fibroblast therapy has been undertaken since 2001. Research has also shown this approach to be safe and well tolerated 36.

Three phases can be recognized in the culture of human dermal fibroblasts: (i) primary cultures established by enzymatic digestion of the dermis or by outgrowth of fibroblasts from explanted tissue pieces; (ii) secondary cultures of actively proliferating cells, provided from passage and expansion of primary cultures; and (iii) terminal cultures reaching a state of replicative senescence and ageing on the cellular level 37, 38. Cells are an attractive *in vitro* system to study transcriptome profiles with respect to their proliferative or secretory activities, not only for studying signatures of ageing 39 but also as dermal fillers in aesthetic dermatology 40. Eca et al. 2012 showed that reimplantation of autologous dermal fibroblasts cultured in medium supplemented with human serum was a viable method with no side effects 41. This method is thus utilized to restore facial fullness and volume after autologous fibroblastic cell reimplantation in both sexes 22, 24, 42, 43.

In our previous study, we documented that autologous fibroblasts of the second passage, in contrast to cells from primary culture, were the optimal population for reimplantation into the dermis of male patients, because they had the highest secretory activity in releasing extracellular matrix components 24. The present study is the continuation of our earlier analysis, in which we focused particularly on the expression of cell cycle-related genes in fibroblasts.

The present study, like the previous one, was performed separately for three passages and the primary culture. Both studies indicate that the optimal population of fibroblasts for reimplantation regarding secretory activity was obtained in the second passage 24. However, analysis of differentially expressed genes showed that the highest number of genes involved in the cell cycle were upregulated in fibroblasts in the third passage (C3) in cluster 5. The results thus indicate that reimplantation of fibroblasts from the third passage could be optimal choice in terms of increasing the number of cells. It has also been shown that cultured fibroblasts isolated from human foreskin had the typical morphology of fibroblasts with high proliferative properties 44.

In our earlier preliminary study, we confirmed that injecting autologous fibroblasts is more beneficial than other methods. We demonstrated that fibroblasts reinjected into donors were accepted and that, three months later, an improvement in the fibrous components of dermal connective tissue could be seen in the morphological analysis 22. In our subsequent study, the optimal population of fibroblasts for reimplantation was characterized by the secretory activity of the cultured cells, reflected by the presence of extracellular matrix components such as elastin and fibrillin 1, and collagen types I and III in a monolayer of cultured cells using microarray analysis 24.

The analysis described in this paper indicates that almost all the expressed genes were directly involved in mechanisms responsible for subsequent events during the cell cycle of dermal fibroblasts of the third passage. The seven genes from cluster 5 that were maximally expressed during G1/S and G2/M were CCN2, CCNB1, MKI67, NCAPG, TOP2A, PLK1, and PRC1. Interestingly, the last of these genes, PRC1, is also involved in the cytokinesis process, as is the ANLN gene. Notably, the profile of cluster 5 also indicated upregulation at the third passage of fibroblasts in genes related to microtubule-dependent processes, namely ASPM, CDC20, CENPE, DLGAP5, KIF-11, KIF-14, KIF-18A, NUSAP1, TPX2, SPAG5, and BUB1, with the last of these activating the spindle checkpoint. Moreover, DNA repair and DNA damage factors known to cooperate in DNA replication were also noted, including FANCD2, encoding Fanconi anemia complex components 45.

Analysis of the fibroblast genes indicated that 21 were involved in the cell cycle, and that one could be characterized as having no function related to the cell cycle. The last gene, *BIRC5*, an inhibitor of cell death, encodes baculoviral IAP repeat containing 5, which promotes cell survival and prevents apoptotic cell death 46. This gene was expressed only during chromosome segregation. Similarly, in nuclear division—the process where the accumulation of upregulated genes was present at the highest level—two of the 21 genes, *ASPM* and *MYBL1*, were specific only to nuclear division.

Transcriptome profiling of human dermal fibroblasts is an attractive model for studying many biological processes, such as proliferative activity, aging, age-dependent transcriptomic changes, and cellular defects, as well as for creating a dataset of fibroblast gene expression, including healthy individuals of various ages 47, and differences between papillary and reticular fibroblasts 48.

The microarray analysis of three passages of male donor dermal fibroblasts performed in this study in relation to the cell cycle can be recognized as a novel basis for potential application in aesthetic medicine.

## Conclusions

The analyses of the activities of genes in cluster 5 suggest that the third passage of dermal fibroblasts exhibits the greatest proliferative activity. Twenty-one out of twenty-two upregulated genes in the third passage of the tested cells were strongly associated with the cell cycle and proliferation. On the other hand, we have previously reported that the second passage of dermal fibroblasts was found to be the best gene profile in terms of extracellular matrix organization. Both fibroblasts taken from the second and the third passage seem to be suitable for skin reimplantation and healing over dermal lesions, and they both have the capacity to improve the skin through different mechanisms. Moreover, the fibroblasts from analyzed passages did not express genes related to aging processes.

The results of this method suggest it as an optimal tool for autologous transplantation, allowing rejuvenation of aging skin, which may be of great importance in the development of aesthetic medicine.

## Study Limitations

In the presented study we have analyzed gene expression profile only in a sample obtained from 46-year-old male. This approach cannot properly reflect the gene expression profile in a general population however it sheds a new light on the mechanisms of cell cycle regulation and proliferation processes in the cell culture.

## Supplementary Material

Supplementary materials.

## Figures and Tables

**Table 1:** Characteristics of primers used in qRT-PCR.

Gene	Primer sequence 5ʹ-3ʹ		Annealing temperature
*ANLN*	forward	GTC GTC AGA TAG AAC CAG CC	59.2 °C
	reverse	CAG AGT GTG TCC CTG CAT TG	
*BUB1*	forward	TAA GGT CAT CTG GGG CTT GG	60.1 °C
	reverse	CCT GGC TCC TGT GGG TTT AT	
BMG*	forward	AAT GCG GCA TCT TCA AAC CT	62.0 °C
	reverse	TGA CTT TGT CAC AGC CCA AGA	
*CDC20*	forward	CTT CGG CTC AGT GGA AAA CC	62.0 °C
	reverse	GGA AGG AAT GTA ACG GCA GG	
*CCNA2*	forward	GGA CCT TCA CCA GAC CTA CC	59.2 °C
	reverse	AGT GTC TCT GGT GGG TTG AG	
*DLGAP5*	forward	CCA TGT CCT TTG GGT CCT CT	62.0 °C
	reverse	GGC TGA GCA ATT CGA CCT TC	
*MKI67*	forward	CCC TAC GGA TTA TAC CTG GCC	62.0 °C
	reverse	CGA CCC CGC TCC TTT TGA TAG	
*PLK1*	forward	TCA TCG AGA CCT CAA GCT GG	60.1 °C
	reverse	CAC AGG GTC TTC TTC CTC TCC	
*PRC1*	forward	GCG CCC TCT TTA CCT ATC CTC	60.1 °C
	reverse	GGC TAA CAC CAA GTC CAC AC	
*SPAG5*	forward	GAC GAT CTG GTG AGA GAG GAG	59.2 °C
	reverse	AGA GAT GGG ACG GAG ATT CC	
*TPX2*	forward	CCA GAC TAC AGG AGG AAG AGC	59.2 °C
	reverse	GGG AGA TAC AGG CAC AGT CAG	

* endogenous control gene

**Table 2:** Upregulated genes involved in the top 5 processes associated with the cell cycle.

GO term:	Upregulated genes
Nuclear division GO: 0000280	ANLN, ASPM*, **BUB1,** CCNA2, **CCNB1**, **CDC20**, **CENPE**, **DLGAP5**, FANCD2, KIF11, KIF14, KIF18A, MKI67, MYBL1*, NCAPG, **NUSAP1**, **PLK1**, PRC1, SPAG5, TOP2A, TPX2
Mitotic nuclear division GO: 0140014	*ANLN, **BUB1,** CCNA2, **CCNB1**, **CDC20**, **CENPE**, **DLGAP5**, KIF11, KIF14, KIF18A, MKI67, NCAPG, **NUSAP1**, **PLK1**, PRC1, SPAG5, TPX2*
Chromosome segregation GO: 0007059	***BUB1, CCNB1, CDC20, CENPE***, D**LGAP5**, FANCD2, KIF14, KIF181, MKI67, NCAPG, **NUSAP1**, **PLK1**, PRC1, SPAG5, TOP2A, BIRC5*
Sister chromatid segregation GO: 0000819	***BUB1, CCNB1, CDC20, CENPE, DLGAP5****, KIF14, KIF18A, NCAPG, **NUSAP1, PLK1**, PRC1, SPAG5, TOP2A*
Regulation of mitotic nuclear division GO: 0007088	*ANLN, **BUB1, CCNA2, CCNB1, CDC20, CENPE, DLGAP5**, KIF11, MKI67, **NUSAP1, PLK1***

**Bolded:** upregulated genes common for all top 5 processes; * upregulated genes present in one process only; GO - Gene Ontology terms

**Table 3:** Summary of all upregulated genes in dermal fibroblasts of 3^rd^ passage

Gene ID (protein)	Ensemble	Function	Ref
ANLN (anilin)	ENSG00000011426	This gene encodes an actin-binding protein that plays a role in cell growth and migration, and in cytokinesis	49
ASPM (abnormal spindle microtubule assembly)	ENSG00000066279	It provides instructions for making a protein that is involved in cell division. This protein is found in cells and tissues throughout the body and it appears to be particularly important for the division of cells in the developing brain.	50
BUB1 (mitotic checkpoint serine/threonine kinase)	ENSG00000169679	The encoded protein functions in part by phosphorylating members of the mitotic checkpoint complex and activating the spindle checkpoint. It also plays a role in inhibiting the activation of the anaphase promoting complex/cyclosome	51, 52
CCNA2 (cyclin A2)	ENSG00000145386	The protein forms complex with cyclin-dependent kinase 2 (Cdk2) to promotes transition through G1/S and G2/M	53
CCNB1 (cyclin B1)	ENSG00000134057	Activated gene product complexes with cdk1 to form the maturation promoting factor (MPF). The protein is essential for G1/S and G2/M phase transitions	54
CDC20 (cell division cycle protein 20)	ENSG00000117399	The protein is active at multiple points in the cell cycle and is required for chromosome separation and mitotic exit.	55
CENPE (centrosome-associated protein E)	ENSG00000138778	Product of the gene is a kinesin-like motor protein that is accumulated in the G2 phase of the cell cycle and is required for stable spindle microtubule capture at kinetochores - necessary step in chromosome alignment during prometaphase	56
DLGAP5 (DLG associated protein 5)	ENSG00000126787	Disks large-associate protein 5 play a unique function in cell cycle and is strongly expressed in all phases of mitosis. It is located at the spindle microtubule, stabilizing spindle formation	57
FANCD2 (FA complementation group D2)	ENSG00000144554	FA complementation group D2 protein facilitates DNA repair and preserve genome stability.	45
KIF11 (kinesin family member 11)	ENSG00000138160	Protein encoded by the gene is responsible for chromosome positioning, centrosome separation and establishing a bipolar spindle during cell mitosis	58
KIF14 (kinesin family member 14)	ENSG00000118193	The product of the gene is included in chromosome segregation, mitotic spindle formation, and cytokinesis.	59
KIF18A (Kinesin family member 18A**)**	ENSG00000121621	The protein is using the hydrolysis of ATP to produce force and movement along microtubules	60
MKI67 Marker of proliferation Ki-67	ENSG00000148773	The protein is necessary for proliferation of cell**.** The highest concentration of mRNA is detected in G2 phase, while protein level increased throughout phases of cell cycle, peaking in M phase. It was suggested that Ki-67 expression is required to organize heterochromatin, thereby controlling gene expression.	61, 62
MYBL1 (MYB proto-oncogene-like 1)	ENSG00000185697	MYBL1 has a key role as a regulator of stem and progenitor cells i.e. in the bone marrow and a neurogenic region of the adult brain.	63
NCAPG (Non-SMC condensin I complex subunit G)	ENSG00000109805	The gene is encoding non-SMC condensing I complex subunit G that is a large protein complex involved in chromosome condensation.	64
NUSAP1 (Nucleolar and spindle associated protein 1)	ENSG00000137804	The gene product is type of a microtubule-associated protein involved in mitotic spindle organization	65
PLK1 (Polo-like kinase 1)	ENSG00000166851	The serine/threonine protein kinase 1 is expressed during the G2/M‐phases of the cell cycle and work in concert with CDK1 and Cyclin B	66
PRC1 (protein regulator of cytokinesis 1)	ENSG00000198901	The protein is required for the completion of cytokinesis at telophase. Its high levels are met during the S and G2/M phases of mitosis but dramatically decrease when the cell exits mitosis and enters the G1 phase	67
SPAG5 (sperm associated antigen 5)	ENSG00000076382	The gene encodes a spindle-binding protein that regulates the assembly timing of the mitotic spindle and the separation of sister chromatids	68, 69
TOP2A (DNA topoisomerase II alpha)	ENSG00000131747	The enzyme, DNA topoisomerase II alpha is required to chromosome condensation, separate the interlinked sister chromatids post-replication	70
TPX2 (TPX2 microtubule nucleation factor)	ENSG00000088325	The protein is involved in stimulating microtubule assembly during mitotic spindle formation	71, 72
BIRC5 (baculoviral IAP repeat containing 5)	ENSG00000089685	The protein is not included directly in cell cycle but promotes cell survival and prevents apoptotic cell death.	46
