# Supplementary material for: Global Gene Expression of Cultured Human Dermal Fibroblasts: Focus on Cell Cycle and Proliferation Status in Improving the Condition of Face Skin
Source: Int J Med Sci. 2021 Feb 3;18(6):1519–31. doi: 10.7150/ijms.46265 (PMC7893558; doi:10.7150/ijms.46265)
Supplement: Supplementary file 1 — Supplementary materials. [file ijmsv18p1519s1.pdf]

| Cluster 1 | | | | | | |
|---|---|---|---|---|---|---|
| | | | **value** | | | |
| | **gene** | **score** | **C0** | **C1** | **C2** | **C3** |
| 1 | A2M | 0.9564367 | -1.2562731 | -0.2261331 | 1.1099019 | 0.3725042 |
| 2 | ABCC3 | 0.9933230 | -1.4700365 | 0.3393595 | 0.7710235 | 0.3596534 |
| 3 | ACTR1B | 0.9698842 | -1.3346546 | 0.1532987 | 1.0893741 | 0.0919818 |
| 4 | ADAMTS6 | 0.9134579 | -1.3016290 | -0.2395719 | 0.5963632 | 0.9448377 |
| 5 | AFF3 | 0.8927700 | -1.3948073 | 0.7855815 | 0.6594343 | -0.0502085 |
| 6 | ALDH1B1 | 0.9557856 | -1.3503262 | 0.3364529 | 1.0310783 | -0.0172049 |
| 7 | ALDH1L2 | 0.9816792 | -1.3637563 | 0.1511891 | 1.0434054 | 0.1691619 |
| 8 | ALG14 | 0.8382430 | -1.3808123 | 0.9511522 | 0.4343328 | -0.0046726 |
| 9 | AMIGO2 | 0.9734952 | -1.3286866 | 0.0818726 | 1.0980733 | 0.1487407 |
| 10 | ANKRD1 | 0.9984583 | -1.4178900 | 0.0858543 | 0.8840288 | 0.4480068 |
| 11 | ARL4D | 0.9731419 | -1.4541501 | 0.1477074 | 0.5816893 | 0.7247535 |
| 12 | ASB6 | 0.9082652 | -1.4537500 | 0.3695812 | 0.2572075 | 0.8269614 |
| 13 | ASNS | 0.8616844 | -1.3681836 | 0.8665773 | 0.6052838 | -0.1036775 |
| 14 | ATP10A | 0.9982447 | -1.4136830 | 0.0852234 | 0.9006986 | 0.4277609 |
| 15 | AVEN | 0.9827392 | -1.3376218 | -0.0583706 | 1.0392903 | 0.3567021 |
| 16 | BAMBI | 0.9676382 | -1.2876158 | -0.1429487 | 1.1015165 | 0.3290481 |
| 17 | BMP6 | 0.9494546 | -1.3780093 | 0.4636035 | 0.9409865 | -0.0265806 |
| 18 | BOK | 0.9835722 | -1.3741743 | 0.1745699 | 1.0250344 | 0.1745699 |
| 19 | C19orf25 | 0.8609682 | -1.3941832 | 0.2831732 | 0.1318927 | 0.9791172 |
| 20 | C1orf198 | 0.9566104 | -1.3234034 | 0.2327194 | 1.0929045 | -0.0022205 |
| 21 | C5orf28 | 0.9055725 | -1.3445774 | 0.6293326 | 0.8773649 | -0.1621201 |
| 22 | C6orf62 | 0.8950345 | -1.2204911 | -0.4082720 | 0.7214273 | 0.9073358 |
| 23 | C7orf60 | 0.9918161 | -1.4152412 | 0.2374195 | 0.9404021 | 0.2374195 |
| 24 | CALD1 | 0.8732032 | -1.4398971 | 0.8648165 | 0.3694847 | 0.2055958 |
| 25 | CD24 | 0.9703728 | -1.3130572 | -0.1963281 | 0.9762938 | 0.5330915 |
| 26 | CDH8 | 0.9929878 | -1.4399955 | 0.2970838 | 0.8756192 | 0.2672925 |
| 27 | CDKL5 | 0.9868311 | -1.3798018 | 0.1590694 | 1.0139440 | 0.2067885 |
| 28 | CEBPG | 0.9786392 | -1.3552425 | 0.1486825 | 1.0578775 | 0.1486825 |
| 29 | CHAC1 | 0.8163095 | -1.2059448 | 0.7247119 | 0.9131852 | -0.4319523 |
| 30 | CLIC3 | 0.9415305 | -1.2166284 | -0.2930749 | 1.1384663 | 0.3712370 |
| 31 | CNN1 | 0.9678340 | -1.2883412 | -0.1514947 | 1.0945260 | 0.3453099 |
| 32 | COL4A1 | 0.9968625 | -1.4047041 | 0.0536484 | 0.9113798 | 0.4396759 |
| 33 | COL4A2 | 0.9876091 | -1.3919030 | 0.2003998 | 0.9911034 | 0.2003998 |
| 34 | COL8A1 | 0.9154305 | -1.3085268 | 0.4763928 | 1.0138061 | -0.1816720 |
| 35 | COMP | 0.8822296 | -1.1181926 | -0.5515550 | 0.9951921 | 0.6745555 |
| 36 | COTL1 | 0.8453310 | -1.3909434 | 0.3477326 | 0.0742722 | 0.9689386 |
| 37 | CSRP1 | 0.9156135 | -1.4625349 | 0.3862005 | 0.2806863 | 0.7956481 |
| 38 | CTGF | 0.8721854 | -1.2188223 | 0.4592025 | 1.0922332 | -0.3326135 |
| 39 | CXorf56 | 0.9533503 | -1.4946633 | 0.4386267 | 0.4386267 | 0.6174099 |
| 40 | DCBLD1 | 0.9357524 | -1.3901844 | -0.0140246 | 0.5004992 | 0.9037097 |

| | | | | | | |
|---|---|---|---|---|---|---|
| 41 | DCLK2 | 0.9317804 | -1.4688119 | 0.3478081 | 0.3445198 | 0.7764840 |
| 42 | DDAH1 | 0.9904537 | -1.4803428 | 0.2792669 | 0.6687415 | 0.5323345 |
| 43 | DIAPH3 | 0.8933553 | -1.2669113 | -0.2956020 | 0.5728922 | 0.9896210 |
| 44 | DSP | 0.9820110 | -1.4247236 | 0.0326442 | 0.7048242 | 0.6872552 |
| 45 | EDN1 | 0.8416584 | -1.4328962 | 0.8837169 | 0.1878319 | 0.3613474 |
| 46 | EIF4EBP1 | 0.9653031 | -1.4601498 | 0.1892278 | 0.5247046 | 0.7462175 |
| 47 | ELN | 0.9883174 | -1.3608053 | -0.0418082 | 0.9866123 | 0.4160012 |
| 48 | ENO1 | 0.8548744 | -1.0647773 | -0.6269834 | 0.9909906 | 0.7007701 |
| 49 | ENTPD4 | 0.9434073 | -1.4838662 | 0.3938586 | 0.3885458 | 0.7014619 |
| 50 | F3 | 0.9913414 | -1.3730123 | 0.0246922 | 0.9945066 | 0.3538135 |
| 51 | FAM101B | 0.9452128 | -1.4837089 | 0.3848101 | 0.3965773 | 0.7023215 |
| 52 | FKBP11 | 0.9023718 | -1.4176344 | 0.2078619 | 0.2767617 | 0.9330108 |
| 53 | FLNB | 0.9723643 | -1.3552042 | 0.2146337 | 1.0534536 | 0.0871169 |
| 54 | FLNC | 0.9731699 | -1.3441944 | -0.1624855 | 0.8792325 | 0.6274473 |
| 55 | FMN2 | 0.9404869 | -1.4777703 | 0.3653031 | 0.3774082 | 0.7350589 |
| 56 | GATA6 | 0.9521678 | -1.2704100 | 0.0346771 | 1.1752434 | 0.0604895 |
| 57 | GFRA1 | 0.9684539 | -1.3042772 | -0.2064093 | 0.9953114 | 0.5153751 |
| 58 | GLS | 0.9405295 | -1.3690826 | 0.4952534 | 0.9361882 | -0.0623590 |
| 59 | GPRC5A | 0.9593856 | -1.2676777 | -0.2332449 | 1.0761958 | 0.4247267 |
| 60 | GRAMD3 | 0.9700947 | -1.3027031 | -0.0284940 | 1.1217289 | 0.2094683 |
| 61 | HAPLN1 | 0.8911967 | -1.1147821 | -0.5190587 | 1.0936161 | 0.5402247 |
| 62 | HAPLN3 | 0.9913387 | -1.4058383 | 0.2094499 | 0.9615651 | 0.2348234 |
| 63 | HIAT1 | 0.9125065 | -1.2441302 | 0.2778614 | 1.1569650 | -0.1906962 |
| 64 | HSPB3 | 0.9075323 | -1.2928153 | -0.2527449 | 0.5852028 | 0.9603574 |
| 65 | HSPB7 | 0.9299202 | -1.4668866 | 0.7046517 | 0.5560584 | 0.2061765 |
| 66 | HSPG2 | 0.9830992 | -1.3394132 | -0.0718440 | 1.0257747 | 0.3854825 |
| 67 | IDH2 | 0.9741665 | -1.3671406 | 0.2434852 | 1.0310509 | 0.0926045 |
| 68 | IFFO2 | 0.9429018 | -1.3113510 | -0.2489629 | 0.7568496 | 0.8034642 |
| 69 | IGHJ3 | 0.9784598 | -1.4597163 | 0.1647122 | 0.6051905 | 0.6898136 |
| 70 | INA | 0.9283863 | -1.1838396 | -0.3458559 | 1.1578639 | 0.3718316 |
| 71 | ITGA1 | 0.9189983 | -1.2479780 | 0.2433272 | 1.1651531 | -0.1605024 |
| 72 | ITGA11 | 0.9298254 | -1.2593846 | 0.2020652 | 1.1666871 | -0.1093676 |
| 73 | ITGA3 | 0.9133571 | -1.3605717 | -0.0580602 | 0.4454831 | 0.9731488 |
| 74 | ITGBL1 | 0.9714782 | -1.3293484 | 0.1105572 | 1.0982125 | 0.1205788 |
| 75 | JAG1 | 0.9539919 | -1.2496056 | -0.1559666 | 1.1644914 | 0.2410807 |
| 76 | KCNE4 | 0.9409939 | -1.2172051 | -0.3090075 | 1.1224554 | 0.4037572 |
| 77 | KCNK6 | 0.8940192 | -1.1452360 | -0.5145357 | 0.9819821 | 0.6777896 |
| 78 | KIAA1462 | 0.9919957 | -1.4002960 | -0.0073922 | 0.8592844 | 0.5484039 |
| 79 | KRTAP1-5 | 0.9685051 | -1.4230772 | 0.0358685 | 0.6181368 | 0.7690718 |
| 80 | KRTAP2-3 | 0.8979487 | -1.3318158 | -0.1124560 | 0.4300231 | 1.0142487 |
| 81 | LARGE | 0.9742919 | -1.3094073 | -0.1320837 | 1.0591670 | 0.3823241 |
| 82 | LGMN | 0.9893626 | -1.4776182 | 0.3824866 | 0.7355707 | 0.3595609 |
| 83 | LINC00862 | 0.8835828 | -1.2202891 | -0.3949935 | 0.6490449 | 0.9662377 |

| | | | | | | |
|---|---|---|---|---|---|---|
| 84 | LINC01023 | 0.9826165 | -1.3703228 | 0.1691729 | 1.0319769 | 0.1691729 |
| 85 | LMOD1 | 0.9809863 | -1.3607686 | 0.1459481 | 1.0484962 | 0.1663242 |
| 86 | LOXL2 | 0.9313672 | -1.2334422 | -0.3785471 | 0.9402843 | 0.6717050 |
| 87 | LPCAT2 | 0.9831972 | -1.3680926 | 0.1522862 | 1.0357110 | 0.1800954 |
| 88 | LYPD6B | 0.9102627 | -1.4736922 | 0.4964845 | 0.2602762 | 0.7169315 |
| 89 | MALL | 0.9157396 | -1.4468997 | 0.2958231 | 0.2958231 | 0.8552535 |
| 90 | MARCH4 | 0.9647904 | -1.4994475 | 0.4620215 | 0.5129268 | 0.5244992 |
| 91 | MFAP5 | 0.9694343 | -1.3004386 | -0.0330413 | 1.1242788 | 0.2092011 |
| 92 | MICAL2 | 0.9859064 | -1.3516373 | -0.0650440 | 0.9958757 | 0.4208057 |
| 93 | MIR614 | 0.9690628 | -1.3105927 | 0.0416170 | 1.1220661 | 0.1469097 |
| 94 | MKNK2 | 0.9782217 | -1.3896923 | 0.2865640 | 0.9864150 | 0.1167133 |
| 95 | MRVI1 | 0.9673376 | -1.2998701 | 0.0010567 | 1.1325622 | 0.1662512 |
| 96 | MTHFD2 | 0.9425479 | -1.3272884 | 0.3566730 | 1.0510120 | -0.0803966 |
| 97 | MYLK | 0.9411237 | -1.2704445 | 0.1502705 | 1.1667149 | -0.0465409 |
| 98 | NEK7 | 0.9748103 | -1.3132629 | -0.1456437 | 1.0376126 | 0.4212940 |
| 99 | NEXN | 0.9000160 | -1.2724591 | 0.4603400 | 1.0538119 | -0.2416928 |
| 100 | NLN | 0.9302125 | -1.3430898 | -0.1531044 | 0.5847416 | 0.9114527 |
| 101 | NOTCH3 | 0.9434055 | -1.2307154 | -0.3234486 | 1.0711973 | 0.4829667 |
| 102 | NPAS2 | 0.9870490 | -1.3866530 | 0.1858079 | 1.0014552 | 0.1993900 |
| 103 | OCLM | 0.8620149 | -1.1192164 | -0.5684548 | 0.8368821 | 0.8507891 |
| 104 | OR2T35 | 0.8451281 | -1.0443143 | -0.6525505 | 0.9966054 | 0.7002594 |
| 105 | OXTR | 0.9695185 | -1.4121410 | -0.0017548 | 0.6508039 | 0.7630918 |
| 106 | PCGF5 | 0.9318590 | -1.4169976 | 0.6484427 | 0.7559638 | 0.0125911 |
| 107 | PCK2 | 0.9447962 | -1.4625153 | 0.6445960 | 0.6445960 | 0.1733232 |
| 108 | PDCD1LG2 | 0.9596792 | -1.3202461 | -0.2250414 | 0.8512253 | 0.6940621 |
| 109 | PDE1C | 0.8956815 | -1.4159835 | 0.2284604 | 0.2490218 | 0.9385014 |
| 110 | PDE5A | 0.9820846 | -1.3473258 | -0.1147672 | 0.9576338 | 0.5044593 |
| 111 | PDLIM5 | 0.9378403 | -1.4808119 | 0.6687172 | 0.5290604 | 0.2830344 |
| 112 | PDLIM7 | 0.9607103 | -1.4996238 | 0.5157107 | 0.5157107 | 0.4682025 |
| 113 | PFKP | 0.8870913 | -1.2288958 | -0.3801079 | 0.6449921 | 0.9640116 |
| 114 | PHGDH | 0.8182102 | -1.4011915 | 0.9718519 | 0.2146698 | 0.2146698 |
| 115 | PKD1 | 0.9786444 | -1.4018931 | 0.3219963 | 0.9570369 | 0.1228600 |
| 116 | PLCB1 | 0.9917511 | -1.4039361 | -0.0025448 | 0.8444188 | 0.5620621 |
| 117 | PLCB4 | 0.9693709 | -1.3105019 | 0.0366376 | 1.1216369 | 0.1522273 |
| 118 | PLEKHA2 | 0.8999955 | -1.4242461 | 0.2497060 | 0.2560618 | 0.9184782 |
| 119 | PLN | 0.8405911 | -1.2159562 | 0.6294031 | 0.9836678 | -0.3971147 |
| 120 | PLOD2 | 0.9454694 | -1.2249168 | -0.2301262 | 1.1680018 | 0.2870412 |
| 121 | PMM2 | 0.9985823 | -1.4316277 | 0.1053098 | 0.8360642 | 0.4902537 |
| 122 | PPME1 | 0.9487660 | -1.2873725 | 0.1537769 | 1.1483927 | -0.0147970 |
| 123 | PPP1R13L | 0.9734964 | -1.3413321 | 0.1440700 | 1.0799593 | 0.1173028 |
| 124 | PRPS1 | 0.9749641 | -1.3653665 | -0.1235581 | 0.8221141 | 0.6668104 |
| 125 | PSAT1 | 0.8972138 | -1.3452782 | 0.6732708 | 0.8415411 | -0.1695337 |
| 126 | PSMD3 | 0.9957093 | -1.4542216 | 0.2950623 | 0.8309288 | 0.3282305 |

| | | | | | | |
|---|---|---|---|---|---|---|
| 127 | RASA4 | 0.9593443 | -1.4998177 | 0.4889166 | 0.4889166 | 0.5219846 |
| 128 | RGS19 | 0.9937487 | -1.4623538 | 0.3300266 | 0.8023007 | 0.3300266 |
| 129 | RNF181 | 0.9941587 | -1.4581018 | 0.3200760 | 0.8179498 | 0.3200760 |
| 130 | RNF187 | 0.9747268 | -1.3100282 | -0.0986180 | 1.0797952 | 0.3288510 |
| 131 | RP11-23E10.2 | 0.9385084 | -1.3313660 | 0.3980990 | 1.0294337 | -0.0961668 |
| 132 | RP11-469A15.2 | 0.9191853 | -1.1784669 | -0.4272957 | 1.0668865 | 0.5388761 |
| 133 | RUSC2 | 0.9689835 | -1.3087624 | -0.2046771 | 0.9803253 | 0.5331142 |
| 134 | SASS6 | 0.8433199 | -1.2385626 | 0.6774082 | 0.9323569 | -0.3712026 |
| 135 | SCUBE3 | 0.9544617 | -1.4477122 | 0.1606622 | 0.4853031 | 0.8017468 |
| 136 | SEL1L3 | 0.9632298 | -1.4636258 | 0.5547788 | 0.7161664 | 0.1926806 |
| 137 | SEMA7A | 0.8668272 | -1.1090822 | -0.5795247 | 0.9090773 | 0.7795295 |
| 138 | SERPINE1 | 0.9614960 | -1.3020981 | -0.2430883 | 0.9332920 | 0.6118944 |
| 139 | SGPL1 | 0.9011777 | -1.2434800 | 0.3560834 | 1.1267890 | -0.2393923 |
| 140 | SH3D21 | 0.8327271 | -1.1081318 | -0.5661594 | 0.6792185 | 0.9950727 |
| 141 | SLC16A3 | 0.9831516 | -1.3867183 | -0.0656461 | 0.8211078 | 0.6312566 |
| 142 | SLC16A4 | 0.9468849 | -1.4873421 | 0.4041130 | 0.4041130 | 0.6791162 |
| 143 | SLC1A4 | 0.9982199 | -1.4274426 | 0.0915608 | 0.8436312 | 0.4922507 |
| 144 | SLC1A5 | 0.9837034 | -1.4790547 | 0.4336760 | 0.7200644 | 0.3253143 |
| 145 | SLC7A5 | 0.9794281 | -1.4374199 | 0.4094733 | 0.8588056 | 0.1691409 |
| 146 | SLC8A1 | 0.9115663 | -1.3670308 | -0.0302760 | 0.4210951 | 0.9762117 |
| 147 | SNAI1 | 0.9165715 | -1.2612318 | -0.3413026 | 0.7351291 | 0.8674053 |
| 148 | SOX9 | 0.9742165 | -1.3784976 | 0.2836875 | 1.0056415 | 0.0891686 |
| 149 | SPDL1 | 0.8542879 | -1.4007999 | 0.3526924 | 0.0973787 | 0.9507288 |
| 150 | SPOCD1 | 0.8582624 | -1.3903723 | 0.2774307 | 0.1260189 | 0.9869227 |
| 151 | SRGN | 0.9400751 | -1.2306321 | -0.3476668 | 1.0332384 | 0.5450605 |
| 152 | SSC5D | 0.9709133 | -1.3845336 | -0.0826021 | 0.7335679 | 0.7335679 |
| 153 | STC2 | 0.8761693 | -1.4532297 | 0.8347700 | 0.3134921 | 0.3049677 |
| 154 | TAF1D | 0.8759385 | -1.3040855 | -0.1407091 | 0.3789979 | 1.0657967 |
| 155 | TECPR2 | 0.9987015 | -1.4221071 | 0.1509933 | 0.9066169 | 0.3644969 |
| 156 | TES | 0.9487457 | -1.2482535 | -0.0545800 | 1.1946463 | 0.1081872 |
| 157 | TGFBI | 0.8974330 | -1.3709095 | 0.0291005 | 0.3395931 | 1.0022159 |
| 158 | TGFBR3L | 0.9704533 | -1.3436383 | 0.1862844 | 1.0737528 | 0.0836012 |
| 159 | TPM1 | 0.8491802 | -1.4366072 | 0.8806786 | 0.2230255 | 0.3329032 |
| 160 | TRAF5 | 0.8623273 | -1.2674946 | 0.6574752 | 0.9275537 | -0.3175343 |
| 161 | TSPAN13 | 0.9309372 | -1.3123717 | -0.2374915 | 0.6743327 | 0.8755306 |
| 162 | TSPAN18 | 0.9996749 | -1.4502356 | 0.2020912 | 0.8203420 | 0.4278024 |
| 163 | TUFT1 | 0.9815601 | -1.3661796 | 0.1634445 | 1.0392907 | 0.1634445 |
| 164 | VASP | 0.9664705 | -1.4741360 | 0.5444934 | 0.6865556 | 0.2430870 |
| 165 | VEGFC | 0.8673465 | -1.3941979 | 0.2522117 | 0.1583583 | 0.9836280 |
| 166 | WWC2 | 0.9910185 | -1.3766313 | -0.0292329 | 0.9435610 | 0.4623032 |
| 167 | YEATS2 | 0.9652886 | -1.3029435 | 0.0495589 | 1.1338390 | 0.1195456 |

| Cluster 2 | | | | | | |
|---|---|---|---|---|---|---|
| | | | **value** | | | |
| | **gene** | **score** | **C0** | **C1** | **C2** | **C3** |
| 1 | C11orf87 | 0.8828397 | -0.9725237 | 1.4008764 | -0.2141763 | -0.2141763 |
| 2 | CNKSR2 | 0.9913703 | -0.1832526 | 1.4323756 | -0.8839496 | -0.3651734 |
| 3 | FCGR1B | 0.9953151 | -0.2964243 | 1.4834578 | -0.6525131 | -0.5345204 |
| 4 | GREM2 | 0.9167843 | -0.5666162 | 1.3102836 | -0.9573174 | 0.2136500 |
| 5 | GSTT2 | 0.9715061 | -0.4453229 | 1.4927260 | -0.4120544 | -0.6353486 |
| 6 | LRRC37A2 | 0.9147700 | -0.6924642 | 1.4561054 | -0.1487151 | -0.6149261 |
| 7 | MIR3191 | 0.9392258 | -0.7941899 | 1.4644567 | -0.3351334 | -0.3351334 |
| 8 | OC90 | 0.8076769 | 0.5003114 | 1.0558635 | -1.2385225 | -0.3176524 |
| 9 | OR4D10 | 0.9620069 | -0.6908257 | 1.4855495 | -0.4047652 | -0.3899587 |
| 10 | OR8H3 | 0.8916834 | -0.9272342 | 1.4186660 | -0.1871028 | -0.3043290 |
| 11 | POTEG | 0.9951495 | -0.4450165 | 1.4956928 | -0.6056598 | -0.4450165 |
| 12 | POTEI | 0.9085706 | -0.7988792 | 1.3460565 | -0.7209266 | 0.1737493 |
| 13 | PPP2R2B | 0.9783114 | -0.5337382 | 1.4357469 | -0.8023905 | -0.0996183 |
| 14 | SLC2A3 | 0.9850127 | -0.5000000 | 1.0500000 | -0.5000000 | -0.5000000 |
| 15 | TWISTNB | 0.9970244 | -0.4285537 | 1.4928297 | -0.6357224 | -0.4285537 |
| 16 | USP17L23 | 0.8738569 | 0.4061643 | 1.2058274 | -1.0081742 | -0.6038175 |
| 17 | ZNF595 | 0.8040890 | 0.1772271 | 1.0356049 | -1.3682009 | 0.1553689 |

| Cluster 3 | | | | | | |
|---|---|---|---|---|---|---|
| | | | value | | | |
| | gene | score | C0 | C1 | C2 | C3 |
| 1 | ABCC9 | 0,8003859 | 1,1622739 | 0,5016627 | -0,9129357 | -0,7510009 |
| 2 | ADH1B | 0,9986559 | 1,4726975 | -0,3972256 | -0,7557428 | -0,3197291 |
| 3 | AKAP12 | 0,9439186 | 1,4039273 | -0,8688878 | -0,5233404 | -0,0116992 |
| 4 | AKR7L | 0,9333496 | 1,4615942 | -0,5448349 | -0,1875720 | -0,7291872 |
| 5 | APCDD1 | 0,9936963 | 1,4556616 | -0,3145686 | -0,8265243 | -0,3145686 |
| 6 | ARMCX1 | 0,8608082 | 1,1795279 | 0,1007674 | -1,2642397 | -0,0160556 |
| 7 | CCL2 | 0,8295384 | 1,3242957 | -1,0352936 | 0,1130324 | -0,4020345 |
| 8 | CCNL1 | 0,9172615 | 1,4018034 | -0,0119922 | -0,5091426 | -0,8806686 |
| 9 | CDC25B | 0,8875107 | 1,2256626 | -0,0009184 | -1,2238258 | -0,0009184 |
| 10 | CDON | 0,9247375 | 1,4509454 | -0,7046982 | -0,1284652 | -0,6177819 |
| 11 | CLIC2 | 0,9850355 | 1,4986310 | -0,4693400 | -0,4693400 | -0,5599509 |
| 12 | CLU | 0,9803218 | 1,4758587 | -0,7104014 | -0,4927477 | -0,2727097 |
| 13 | COL14A1 | 0,9667152 | 1,4794880 | -0,7232215 | -0,3457586 | -0,4105080 |
| 14 | COLEC12 | 0,9643067 | 1,4865840 | -0,4652409 | -0,3493521 | -0,6719910 |
| 15 | CTAGE1 | 0,8711616 | 1,2389673 | 0,3008336 | -1,0772810 | -0,4625199 |
| 16 | CTSK | 0,8366557 | 1,2396451 | 0,3822796 | -0,7807750 | -0,8411497 |
| 17 | CTSL | 0,9200392 | 1,4105423 | -0,9507548 | -0,2490450 | -0,2107426 |
| 18 | DCLK1 | 0,9632814 | 1,4817079 | -0,3786498 | -0,3890521 | -0,7140060 |
| 19 | DCN | 0,9856063 | 1,4993334 | -0,5208558 | -0,4576219 | -0,5208558 |
| 20 | DHCR24 | 0,8488743 | 1,2240183 | -0,9729064 | -0,6372706 | 0,3861587 |
| 21 | DKFZP434I0714 | 0,8805552 | 1,3280446 | 0,1991915 | -0,6403393 | -0,8868968 |
| 22 | DNM1 | 0,9670216 | 1,4057136 | -0,6448941 | -0,7795699 | 0,0187504 |
| 23 | EEPD1 | 0,9843658 | 1,4401760 | -0,1925548 | -0,8589025 | -0,3887187 |
| 24 | EGFR | 0,9837185 | 1,4282757 | -0,4853184 | -0,8455844 | -0,0973729 |
| 25 | FADS1 | 0,9977000 | 1,4714665 | -0,3532247 | -0,7650171 | -0,3532247 |
| 26 | FAP | 0,8884338 | 1,4099750 | -0,8055459 | -0,0018821 | -0,6025470 |
| 27 | FASN | 0,8665930 | 1,1936569 | -0,4903871 | -1,0889983 | 0,3857284 |
| 28 | FBLN7 | 0,8030529 | 1,2837591 | -1,1077966 | 0,1457578 | -0,3217203 |
| 29 | FLT3LG | 0,9624297 | 1,3975732 | -0,0482628 | -0,9336344 | -0,4156760 |
| 30 | FOSB | 0,9577609 | 1,4230998 | -0,0323359 | -0,7529537 | -0,6378103 |
| 31 | GH2 | 0,8771708 | 1,2411684 | 0,2646828 | -1,1104519 | -0,3953993 |
| 32 | GLUD1 | 0,9020424 | 1,3077533 | -0,8776376 | -0,6771368 | 0,2470211 |
| 33 | GPNMB | 0,9366857 | 1,4087353 | 0,0116451 | -0,6527933 | -0,7675871 |
| 34 | HCG9 | 0,9940026 | 1,4532429 | -0,3831518 | -0,8255040 | -0,2445872 |
| 35 | IER2 | 0,9499762 | 1,3559480 | -0,0660832 | -1,0481460 | -0,2417188 |
| 36 | IFI16 | 0,9662324 | 1,4171821 | -0,0561028 | -0,8568616 | -0,5042177 |
| 37 | IFIT3 | 0,9979277 | 1,4942416 | -0,5043359 | -0,6020293 | -0,3878764 |
| 38 | IFITM1 | 0,9497168 | 1,4731927 | -0,6849376 | -0,2361866 | -0,5520685 |
| 39 | IGKV1-16 | 0,9348396 | 1,4514556 | -0,2892269 | -0,3220421 | -0,8401866 |
| 40 | IGSF10 | 0,9901047 | 01,05,2020 | -0,5000000 | -0,5000000 | -0,5000000 |

| | | | | | | |
|---|---|---|---|---|---|---|
| 41 | IL13RA2 | 0,9972474 | 1,4937199 | -0,4075558 | -0,6231537 | -0,4630104 |
| 42 | JUN | 0,8959453 | 1,3716770 | 0,0590721 | -0,5014923 | -0,9292568 |
| 43 | JUNB | 0,9110896 | 1,3136049 | 0,1832135 | -0,9940127 | -0,5028057 |
| 44 | KCND2 | 0,9462754 | 1,4708264 | -0,4379242 | -0,2803913 | -0,7525110 |
| 45 | KLF4 | 0,9838206 | 1,4980366 | -0,4631778 | -0,4631778 | -0,5716809 |
| 46 | KLF6 | 0,9370906 | 1,3353178 | 0,0135313 | -1,0662449 | -0,2826041 |
| 47 | KRTAP10-5 | 0,9406814 | 1,4660233 | -0,4436397 | -0,2549444 | -0,7674392 |
| 48 | LDLR | 0,8249128 | 1,1111791 | 0,1045996 | -1,3203783 | 0,1045996 |
| 49 | LGALS9B | 0,9445303 | 1,3821783 | 0,0548283 | -0,8829464 | -0,5540602 |
| 50 | LRIG3 | 0,9622602 | 1,4840118 | -0,4371380 | -0,3521960 | -0,6946778 |
| 51 | LRRC32 | 0,9964308 | 1,4882064 | -0,3556195 | -0,6595674 | -0,4730194 |
| 52 | LRRN4CL | 0,9719891 | 1,4816749 | -0,7142742 | -0,3837003 | -0,3837003 |
| 53 | MAFB | 0,9985090 | 1,4759278 | -0,3658041 | -0,7443197 | -0,3658041 |
| 54 | MCL1 | 0,9300157 | 1,3951364 | 0,0517973 | -0,6781745 | -0,7687592 |
| 55 | MIR3620 | 0,8534974 | 1,2173284 | 0,3626198 | -1,0531283 | -0,5268199 |
| 56 | MMP1 | 0,9859884 | 1,4967153 | -0,4163871 | -0,5020164 | -0,5783118 |
| 57 | MYLIP | 0,9433502 | 1,3669880 | 0,0521119 | -0,9562337 | -0,4628662 |
| 58 | NAV2 | 0,9985003 | 1,4727394 | -0,3829505 | -0,7576945 | -0,3320944 |
| 59 | NPC2 | 0,9197807 | 1,3922811 | 0,0439252 | -0,5991922 | -0,8370141 |
| 60 | OAF | 0,8538230 | 1,3309059 | -1,0918448 | -0,0563266 | -0,1827345 |
| 61 | OR2A14 | 0,9843459 | 1,4345964 | -0,2122414 | -0,8848660 | -0,3374890 |
| 62 | OR2L3 | 0,9716015 | 1,3932496 | -0,3023985 | -0,9769535 | -0,1138976 |
| 63 | OR56A5 | 0,9894255 | 1,4973952 | -0,4167941 | -0,5293259 | -0,5512752 |
| 64 | OSR2 | 0,9086790 | 1,3620951 | 0,1343899 | -0,6894936 | -0,8069913 |
| 65 | OXT | 0,9819089 | 1,4864710 | -0,6755045 | -0,4567874 | -0,3541792 |
| 66 | PDE7B | 0,9694496 | 1,3938353 | -0,4796165 | -0,9094882 | -0,0047305 |
| 67 | PDGFRL | 0,9901047 | 01,05,2020 | -0,5000000 | -0,5000000 | -0,5000000 |
| 68 | PHLDA1 | 0,8629283 | 1,1799823 | 0,0432353 | -1,2664530 | 0,0432353 |
| 69 | PPAP2B | 0,9536739 | 1,4728103 | -0,3569138 | -0,3569138 | -0,7589827 |
| 70 | PPARGC1A | 0,9607123 | 1,4441186 | -0,1001836 | -0,6454700 | -0,6984650 |
| 71 | PRSS36 | 0,9468640 | 1,4133100 | -0,8672163 | -0,4981930 | -0,0479006 |
| 72 | PTGFR | 0,9949765 | 1,4893338 | -0,5768669 | -0,5843183 | -0,3281486 |
| 73 | PTGS2 | 0,9962516 | 1,4830948 | -0,3309499 | -0,6926551 | -0,4594898 |
| 74 | REV3L | 0,9361165 | 1,4474530 | -0,2322858 | -0,3702994 | -0,8448678 |
| 75 | RNPS1 | 0,8743238 | 1,3044197 | -1,0566237 | -0,3989745 | 0,1511785 |
| 76 | RRP7B | 0,9583549 | 1,4586448 | -0,1844671 | -0,5218436 | -0,7523341 |
| 77 | RSPO3 | 0,9803763 | 1,4567408 | -0,1653764 | -0,7371862 | -0,5541782 |
| 78 | SAMHD1 | 0,9626130 | 1,4814892 | -0,3830851 | -0,3830851 | -0,7153190 |
| 79 | SAT1 | 0,9952904 | 1,4789827 | -0,3084349 | -0,7125276 | -0,4580202 |
| 80 | SCD | 0,8035994 | 1,0801035 | -0,4529406 | -1,1596177 | 0,5324548 |
| 81 | SCPEP1 | 0,9677274 | 1,4813957 | -0,3320770 | -0,4428927 | -0,7064260 |
| 82 | SLC39A8 | 0,9353952 | 1,4535844 | -0,3099859 | -0,3099859 | -0,8336126 |
| 83 | SLC43A3 | 0,9391875 | 1,3226945 | -0,2864100 | -1,0800612 | 0,0437766 |

| | | | | | | |
|---|---|---|---|---|---|---|
| 84 | SLC7A8 | 0,9896718 | 1,4396441 | -0,3689531 | -0,8655794 | -0,2051116 |
| 85 | SLC9A9 | 0,9461152 | 1,4545222 | -0,2201760 | -0,4247119 | -0,8096342 |
| 86 | SNORA70 | 0,9224294 | 1,3932171 | 0,0464464 | -0,6186550 | -0,8210085 |
| 87 | SREBF1 | 0,9008440 | 1,3654761 | -1,0191763 | -0,3087833 | -0,0375164 |
| 88 | SRSF7 | 0,9905075 | 1,4999933 | -0,4978825 | -0,5042283 | -0,4978825 |
| 89 | SVIL | 0,9626999 | 1,4834485 | -0,4174135 | -0,3642997 | -0,7017352 |
| 90 | TBC1D29 | 0,9929005 | 1,4731216 | -0,2726362 | -0,7325294 | -0,4679561 |
| 91 | TCEAL3 | 0,9751882 | 1,4840329 | -0,7000683 | -0,4031764 | -0,3807881 |
| 92 | TGFBR3 | 0,9832895 | 1,4720554 | -0,2227644 | -0,6637914 | -0,5854996 |
| 93 | TNFSF10 | 0,9901047 | 1,0500000 | -0,5000000 | -0,5000000 | -0,5000000 |
| 94 | UBC | 0,9877054 | 1,4894297 | -0,3288813 | -0,5802742 | -0,5802742 |
| 95 | VCX3B | 0,8789211 | 1,3320112 | -1,0700008 | -0,2835173 | 0,0215069 |
| 96 | WISP2 | 0,9369645 | 1,4513546 | -0,2656637 | -0,3478342 | -0,8378568 |
| 97 | ZCCHC14 | 0,9053322 | 1,2654276 | 0,0412018 | -1,1745417 | -0,1320877 |
| 98 | ZFP36 | 0,8685210 | 1,1862485 | -0,0807513 | -1,2510436 | 0,1455464 |
| 99 | ZNF14 | 0,9249957 | 1,4401905 | -0,8389250 | -0,1569383 | -0,4443272 |
| 100 | ZNF436 | 0,9718644 | 1,4754005 | -0,7418035 | -0,4106090 | -0,3229880 |
| 101 | ZNF480 | 0,9266371 | 1,3714028 | 0,1124768 | -0,7951091 | -0,6887705 |
| 102 | ZNF563 | 0,9377898 | 1,4582201 | -0,3447016 | -0,2971314 | -0,8163871 |

| Cluster 4 | | | | | | |
|---|---|---|---|---|---|---|
| | | | **value** | | | |
| | **gene** | **score** | **C0** | **C1** | **C2** | **C3** |
| 1 | ACAN | 0,9649215 | 0,9649215 | 0,9649215 | 0,9649215 | 0,9649215 |
| 2 | ADAMTS12 | 0,8833638 | 0,8833638 | 0,8833638 | 0,8833638 | 0,8833638 |
| 3 | AKAP6 | 0,9703476 | 0,9703476 | 0,9703476 | 0,9703476 | 0,9703476 |
| 4 | ANGPTL4 | 0,9872129 | 0,9872129 | 0,9872129 | 0,9872129 | 0,9872129 |
| 5 | ASPN | 0,9974152 | 0,9974152 | 0,9974152 | 0,9974152 | 0,9974152 |
| 6 | B3GALT2 | 0,9456934 | 0,9456934 | 0,9456934 | 0,9456934 | 0,9456934 |
| 7 | BAIAP2L1 | 0,9709610 | 0,9709610 | 0,9709610 | 0,9709610 | 0,9709610 |
| 8 | BNIP3 | 0,9180323 | 0,9180323 | 0,9180323 | 0,9180323 | 0,9180323 |
| 9 | CD55 | 0,9715522 | 0,9715522 | 0,9715522 | 0,9715522 | 0,9715522 |
| 10 | CEP112 | 0,9957547 | 0,9957547 | 0,9957547 | 0,9957547 | 0,9957547 |
| 11 | COL11A1 | 0,9787672 | 0,9787672 | 0,9787672 | 0,9787672 | 0,9787672 |
| 12 | COL12A1 | 0,9623294 | 0,9623294 | 0,9623294 | 0,9623294 | 0,9623294 |
| 13 | COL5A1 | 0,8803192 | 0,8803192 | 0,8803192 | 0,8803192 | 0,8803192 |
| 14 | COL5A2 | 0,9939026 | 0,9939026 | 0,9939026 | 0,9939026 | 0,9939026 |
| 15 | COL8A2 | 0,9491913 | 0,9491913 | 0,9491913 | 0,9491913 | 0,9491913 |
| 16 | CORO2B | 0,9938074 | 0,9938074 | 0,9938074 | 0,9938074 | 0,9938074 |
| 17 | CPEB2 | 0,9327377 | 0,9327377 | 0,9327377 | 0,9327377 | 0,9327377 |
| 18 | DAAM1 | 0,9990185 | 0,9990185 | 0,9990185 | 0,9990185 | 0,9990185 |
| 19 | DACT1 | 0,9814924 | 0,9814924 | 0,9814924 | 0,9814924 | 0,9814924 |
| 20 | ECM2 | 0,9491913 | 0,9491913 | 0,9491913 | 0,9491913 | 0,9491913 |
| 21 | EDIL3 | 0,9534272 | 0,9534272 | 0,9534272 | 0,9534272 | 0,9534272 |
| 22 | ERCC6 | 0,9996227 | 0,9996227 | 0,9996227 | 0,9996227 | 0,9996227 |
| 23 | ERRFI1 | 0,9631963 | 0,9631963 | 0,9631963 | 0,9631963 | 0,9631963 |
| 24 | FAM219A | 0,9352384 | 0,9352384 | 0,9352384 | 0,9352384 | 0,9352384 |
| 25 | FAM66C | 0,9902598 | 0,9902598 | 0,9902598 | 0,9902598 | 0,9902598 |
| 26 | FAT1 | 0,9381797 | 0,9381797 | 0,9381797 | 0,9381797 | 0,9381797 |
| 27 | FHOD3 | 0,9768044 | 0,9768044 | 0,9768044 | 0,9768044 | 0,9768044 |
| 28 | FLG | 0,9991171 | 0,9991171 | 0,9991171 | 0,9991171 | 0,9991171 |
| 29 | FNDC1 | 0,9778295 | 0,9778295 | 0,9778295 | 0,9778295 | 0,9778295 |
| 30 | FOXG1 | 0,9962603 | 0,9962603 | 0,9962603 | 0,9962603 | 0,9962603 |
| 31 | FRY | 0,9352480 | 0,9352480 | 0,9352480 | 0,9352480 | 0,9352480 |
| 32 | GCLM | 0,9974824 | 0,9974824 | 0,9974824 | 0,9974824 | 0,9974824 |
| 33 | GCNT4 | 0,9282243 | 0,9282243 | 0,9282243 | 0,9282243 | 0,9282243 |
| 34 | GOLGA8A | 0,9878542 | 0,9878542 | 0,9878542 | 0,9878542 | 0,9878542 |
| 35 | GPC4 | 0,9027848 | 0,9027848 | 0,9027848 | 0,9027848 | 0,9027848 |
| 36 | GPR155 | 0,9363016 | 0,9363016 | 0,9363016 | 0,9363016 | 0,9363016 |
| 37 | GYS1 | 0,9203245 | 0,9203245 | 0,9203245 | 0,9203245 | 0,9203245 |
| 38 | HAS2 | 0,8638688 | 0,8638688 | 0,8638688 | 0,8638688 | 0,8638688 |
| 39 | HMCN1 | 0,9893018 | 0,9893018 | 0,9893018 | 0,9893018 | 0,9893018 |
| 40 | HSPA2 | 0,9959488 | 0,9959488 | 0,9959488 | 0,9959488 | 0,9959488 |

| | | | | | | |
|---|---|---|---|---|---|---|
| 41 | HTR2A | 0,9679243 | 0,9679243 | 0,9679243 | 0,9679243 | 0,9679243 |
| 42 | HUNK | 0,9814817 | 0,9814817 | 0,9814817 | 0,9814817 | 0,9814817 |
| 43 | IGFBP5 | 0,8729157 | 0,8729157 | 0,8729157 | 0,8729157 | 0,8729157 |
| 44 | IL20RB | 0,9676197 | 0,9676197 | 0,9676197 | 0,9676197 | 0,9676197 |
| 45 | IMPA2 | 0,9401067 | 0,9401067 | 0,9401067 | 0,9401067 | 0,9401067 |
| 46 | INHBB | 0,9030614 | 0,9030614 | 0,9030614 | 0,9030614 | 0,9030614 |
| 47 | INIP | 0,9603939 | 0,9603939 | 0,9603939 | 0,9603939 | 0,9603939 |
| 48 | ITGA6 | 0,9558884 | 0,9558884 | 0,9558884 | 0,9558884 | 0,9558884 |
| 49 | ITGA8 | 0,9835958 | 0,9835958 | 0,9835958 | 0,9835958 | 0,9835958 |
| 50 | IVNS1ABP | 0,9710722 | 0,9710722 | 0,9710722 | 0,9710722 | 0,9710722 |
| 51 | JADE1 | 0,9848787 | 0,9848787 | 0,9848787 | 0,9848787 | 0,9848787 |
| 52 | JPH2 | 0,9873974 | 0,9873974 | 0,9873974 | 0,9873974 | 0,9873974 |
| 53 | KCTD20 | 0,9754608 | 0,9754608 | 0,9754608 | 0,9754608 | 0,9754608 |
| 54 | KRTAP1-1 | 0,8845501 | 0,8845501 | 0,8845501 | 0,8845501 | 0,8845501 |
| 55 | LEP | 0,9897012 | 0,9897012 | 0,9897012 | 0,9897012 | 0,9897012 |
| 56 | LGR5 | 0,9402961 | 0,9402961 | 0,9402961 | 0,9402961 | 0,9402961 |
| 57 | LINC00312 | 0,9973279 | 0,9973279 | 0,9973279 | 0,9973279 | 0,9973279 |
| 58 | LMCD1 | 0,9850230 | 0,9850230 | 0,9850230 | 0,9850230 | 0,9850230 |
| 59 | LUZP2 | 0,9299540 | 0,9299540 | 0,9299540 | 0,9299540 | 0,9299540 |
| 60 | MAMDC2 | 0,9832126 | 0,9832126 | 0,9832126 | 0,9832126 | 0,9832126 |
| 61 | MCAM | 0,9818183 | 0,9818183 | 0,9818183 | 0,9818183 | 0,9818183 |
| 62 | MEG3 | 0,8470589 | 0,8470589 | 0,8470589 | 0,8470589 | 0,8470589 |
| 63 | MEGF6 | 0,9018239 | 0,9018239 | 0,9018239 | 0,9018239 | 0,9018239 |
| 64 | MFGE8 | 0,9998504 | 0,9998504 | 0,9998504 | 0,9998504 | 0,9998504 |
| 65 | MIR27B | 0,9442481 | 0,9442481 | 0,9442481 | 0,9442481 | 0,9442481 |
| 66 | MIR4640 | 0,9532481 | 0,9532481 | 0,9532481 | 0,9532481 | 0,9532481 |
| 67 | MSTN | 0,9832979 | 0,9832979 | 0,9832979 | 0,9832979 | 0,9832979 |
| 68 | MUC1 | 0,9000650 | 0,9000650 | 0,9000650 | 0,9000650 | 0,9000650 |
| 69 | MYH10 | 0,9309460 | 0,9309460 | 0,9309460 | 0,9309460 | 0,9309460 |
| 70 | MYH11 | 0,9210172 | 0,9210172 | 0,9210172 | 0,9210172 | 0,9210172 |
| 71 | MYH2 | 0,9798202 | 0,9798202 | 0,9798202 | 0,9798202 | 0,9798202 |
| 72 | NALCN | 0,9761917 | 0,9761917 | 0,9761917 | 0,9761917 | 0,9761917 |
| 73 | NEDD1 | 0,9398376 | 0,9398376 | 0,9398376 | 0,9398376 | 0,9398376 |
| 74 | NFASC | 0,9797447 | 0,9797447 | 0,9797447 | 0,9797447 | 0,9797447 |
| 75 | NUAK1 | 0,9565002 | 0,9565002 | 0,9565002 | 0,9565002 | 0,9565002 |
| 76 | P4HA1 | 0,8568481 | 0,8568481 | 0,8568481 | 0,8568481 | 0,8568481 |
| 77 | PACSIN3 | 0,9715678 | 0,9715678 | 0,9715678 | 0,9715678 | 0,9715678 |
| 78 | PADI2 | 0,9733506 | 0,9733506 | 0,9733506 | 0,9733506 | 0,9733506 |
| 79 | PDE11A | 0,9941237 | 0,9941237 | 0,9941237 | 0,9941237 | 0,9941237 |
| 80 | PDK1 | 0,8975501 | 0,8975501 | 0,8975501 | 0,8975501 | 0,8975501 |
| 81 | PGK1 | 0,9869258 | 0,9869258 | 0,9869258 | 0,9869258 | 0,9869258 |
| 82 | PHKA1 | 0,9920712 | 0,9920712 | 0,9920712 | 0,9920712 | 0,9920712 |
| 83 | PKP4 | 0,9904189 | 0,9904189 | 0,9904189 | 0,9904189 | 0,9904189 |

| | | | | | | |
|---|---|---|---|---|---|---|
| 84 | PLOD1 | 0,9167084 | 0,9167084 | 0,9167084 | 0,9167084 | 0,9167084 |
| 85 | POLR2J | 0,9970975 | 0,9970975 | 0,9970975 | 0,9970975 | 0,9970975 |
| 86 | PPP1R14A | 0,9640831 | 0,9640831 | 0,9640831 | 0,9640831 | 0,9640831 |
| 87 | PTGIS | 0,8553227 | 0,8553227 | 0,8553227 | 0,8553227 | 0,8553227 |
| 88 | PTPRB | 0,9837575 | 0,9837575 | 0,9837575 | 0,9837575 | 0,9837575 |
| 89 | PVR | 0,9666544 | 0,9666544 | 0,9666544 | 0,9666544 | 0,9666544 |
| 90 | RAPH1 | 0,9736981 | 0,9736981 | 0,9736981 | 0,9736981 | 0,9736981 |
| 91 | RASGRP1 | 0,9718980 | 0,9718980 | 0,9718980 | 0,9718980 | 0,9718980 |
| 92 | RASSF2 | 0,9594532 | 0,9594532 | 0,9594532 | 0,9594532 | 0,9594532 |
| 93 | RAVER2 | 0,9608269 | 0,9608269 | 0,9608269 | 0,9608269 | 0,9608269 |
| 94 | RDH10 | 0,9488981 | 0,9488981 | 0,9488981 | 0,9488981 | 0,9488981 |
| 95 | RGCC | 0,9242260 | 0,9242260 | 0,9242260 | 0,9242260 | 0,9242260 |
| 96 | RIMS1 | 0,9539484 | 0,9539484 | 0,9539484 | 0,9539484 | 0,9539484 |
| 97 | RNF144B | 0,9887346 | 0,9887346 | 0,9887346 | 0,9887346 | 0,9887346 |
| 98 | RNF217 | 0,9655031 | 0,9655031 | 0,9655031 | 0,9655031 | 0,9655031 |
| 99 | ROR1 | 0,9491913 | 0,9491913 | 0,9491913 | 0,9491913 | 0,9491913 |
| 100 | SCN9A | 0,9913465 | 0,9913465 | 0,9913465 | 0,9913465 | 0,9913465 |
| 101 | SDC2 | 0,9890486 | 0,9890486 | 0,9890486 | 0,9890486 | 0,9890486 |
| 102 | SDPR | 0,9804184 | 0,9804184 | 0,9804184 | 0,9804184 | 0,9804184 |
| 103 | SFRP4 | 0,9432725 | 0,9432725 | 0,9432725 | 0,9432725 | 0,9432725 |
| 104 | SGCD | 0,9974242 | 0,9974242 | 0,9974242 | 0,9974242 | 0,9974242 |
| 105 | SLC25A4 | 0,9994171 | 0,9994171 | 0,9994171 | 0,9994171 | 0,9994171 |
| 106 | SLC2A1 | 0,9635234 | 0,9635234 | 0,9635234 | 0,9635234 | 0,9635234 |
| 107 | SLC38A4 | 0,9491913 | 0,9491913 | 0,9491913 | 0,9491913 | 0,9491913 |
| 108 | SLC4A4 | 0,9366716 | 0,9366716 | 0,9366716 | 0,9366716 | 0,9366716 |
| 109 | SNORD19B | 0,9970976 | 0,9970976 | 0,9970976 | 0,9970976 | 0,9970976 |
| 110 | SORBS1 | 0,9545951 | 0,9545951 | 0,9545951 | 0,9545951 | 0,9545951 |
| 111 | SORBS2 | 0,9604649 | 0,9604649 | 0,9604649 | 0,9604649 | 0,9604649 |
| 112 | SORT1 | 0,9132718 | 0,9132718 | 0,9132718 | 0,9132718 | 0,9132718 |
| 113 | SSH1 | 0,9569616 | 0,9569616 | 0,9569616 | 0,9569616 | 0,9569616 |
| 114 | SYNPO2 | 0,8532369 | 0,8532369 | 0,8532369 | 0,8532369 | 0,8532369 |
| 115 | SYTL2 | 0,9804600 | 0,9804600 | 0,9804600 | 0,9804600 | 0,9804600 |
| 116 | TEAD3 | 0,9538625 | 0,9538625 | 0,9538625 | 0,9538625 | 0,9538625 |
| 117 | TGFBR1 | 0,9844837 | 0,9844837 | 0,9844837 | 0,9844837 | 0,9844837 |
| 118 | TGM2 | 0,9853114 | 0,9853114 | 0,9853114 | 0,9853114 | 0,9853114 |
| 119 | TM4SF20 | 0,9874997 | 0,9874997 | 0,9874997 | 0,9874997 | 0,9874997 |
| 120 | TMEM130 | 0,9991039 | 0,9991039 | 0,9991039 | 0,9991039 | 0,9991039 |
| 121 | TMEM45A | 0,9747412 | 0,9747412 | 0,9747412 | 0,9747412 | 0,9747412 |
| 122 | TNFSF4 | 0,9998756 | 0,9998756 | 0,9998756 | 0,9998756 | 0,9998756 |
| 123 | TRAM1 | 0,9746942 | 0,9746942 | 0,9746942 | 0,9746942 | 0,9746942 |
| 124 | TRIB3 | 0,9201547 | 0,9201547 | 0,9201547 | 0,9201547 | 0,9201547 |
| 125 | TSPAN2 | 0,9950350 | 0,9950350 | 0,9950350 | 0,9950350 | 0,9950350 |
| 126 | TTLL7 | 0,9847606 | 0,9847606 | 0,9847606 | 0,9847606 | 0,9847606 |

| | | | | | | |
|---|---|---|---|---|---|---|
| 127 | UNC5B | 0,9258421 | 0,9258421 | 0,9258421 | 0,9258421 | 0,9258421 |
| 128 | USP53 | 0,9930812 | 0,9930812 | 0,9930812 | 0,9930812 | 0,9930812 |
| 129 | VCAN | 0,9745706 | 0,9745706 | 0,9745706 | 0,9745706 | 0,9745706 |
| 130 | VEGFA | 0,9991300 | 0,9991300 | 0,9991300 | 0,9991300 | 0,9991300 |
| 131 | VLDLR | 0,9880776 | 0,9880776 | 0,9880776 | 0,9880776 | 0,9880776 |
| 132 | WARS | 0,8938790 | 0,8938790 | 0,8938790 | 0,8938790 | 0,8938790 |
| 133 | WFDC1 | 0,9550107 | 0,9550107 | 0,9550107 | 0,9550107 | 0,9550107 |
| 134 | WIPF3 | 0,9503610 | 0,9503610 | 0,9503610 | 0,9503610 | 0,9503610 |
| 135 | WISP1 | 0,9901755 | 0,9901755 | 0,9901755 | 0,9901755 | 0,9901755 |
| 136 | WNT2 | 0,9328079 | 0,9328079 | 0,9328079 | 0,9328079 | 0,9328079 |
| 137 | WNT5A | 0,9389367 | 0,9389367 | 0,9389367 | 0,9389367 | 0,9389367 |
| 138 | ZNF699 | 0,9756463 | 0,9756463 | 0,9756463 | 0,9756463 | 0,9756463 |

| Cluster 5 | | | | | | |
|---|---|---|---|---|---|---|
| | | | **value** | | | |
| | **gene** | **score** | **C0** | **C1** | **C2** | **C3** |
| 1 | ACTN1 | 0,8568832 | -1,3507554 | 0,1171616 | 0,1691050 | 1,0644889 |
| 2 | ADAM23 | 0,9528242 | -1,1432622 | 0,1029451 | -0,2352300 | 1,2755472 |
| 3 | ADIRF | 0,8531615 | -1,3576651 | 0,1842304 | 0,1207075 | 1,0527272 |
| 4 | ALCAM | 0,8461209 | -1,1077886 | -0,4996627 | 0,4637316 | 1,1437197 |
| 5 | ANKRD54 | 0,9382882 | -0,5520305 | -0,0087982 | -0,8460670 | 1,4068957 |
| 6 | ANLN | 0,9611408 | -0,4482156 | -0,4482156 | -0,5997374 | 1,4961686 |
| 7 | ARHGAP11A | 0,9841218 | -1,0156900 | -0,0414513 | -0,3104412 | 1,3675825 |
| 8 | ARNTL2 | 0,9491641 | -1,0416333 | -0,3891543 | 0,1071249 | 1,3236628 |
| 9 | ARSJ | 0,9284104 | -1,1844692 | -0,1911361 | 0,1335669 | 1,2420384 |
| 10 | ASPM | 0,9358467 | -0,3314613 | -0,5070302 | -0,6488179 | 1,4873094 |
| 11 | BHLHE40 | 0,9134637 | -1,2311945 | -0,1254788 | 0,1548226 | 1,2018507 |
| 12 | BIRC5 | 0,9844658 | -0,7522883 | -0,5073138 | -0,2018829 | 1,4614850 |
| 13 | BLID | 0,8930357 | -1,2107154 | -0,2494901 | 0,2796459 | 1,1805596 |
| 14 | BUB1 | 0,9695554 | -0,5000000 | -0,5000000 | -0,5000000 | 1,5000000 |
| 15 | C12orf75 | 0,9841873 | -1,0300584 | -0,1674527 | -0,1738772 | 1,3713883 |
| 16 | CASC5 | 0,9126248 | -0,2899557 | -0,3055890 | -0,8522721 | 1,4478168 |
| 17 | CCNA2 | 0,9743771 | -0,6090664 | -0,1711028 | -0,6812300 | 1,4613992 |
| 18 | CCNB1 | 0,9260775 | -0,3069550 | -0,6423264 | -0,5359600 | 1,4852415 |
| 19 | CDA | 0,9625728 | -0,9906740 | -0,4097972 | 0,0407011 | 1,3597701 |
| 20 | CDC20 | 0,9463451 | -0,4494923 | -0,2188557 | -0,7898176 | 1,4581656 |
| 21 | CDH2 | 0,8328054 | -1,3539720 | 0,3849300 | -0,0394386 | 1,0084806 |
| 22 | CDK1 | 0,9613513 | -0,4489895 | -0,4585947 | -0,5893389 | 1,4969232 |
| 23 | CENPE | 0,9611038 | -0,4486144 | -0,4380056 | -0,6087947 | 1,4954147 |
| 24 | CEP55 | 0,9594265 | -0,4392084 | -0,4787077 | -0,5794663 | 1,4973823 |
| 25 | CGB8 | 0,8840545 | -0,2308741 | -0,2308741 | -0,9495472 | 1,4112953 |
| 26 | CKAP2 | 0,9684627 | -0,4912886 | -0,4912886 | -0,5173100 | 1,4998871 |
| 27 | CKAP2L | 0,9529380 | -0,4129379 | -0,4129379 | -0,6636143 | 1,4894901 |
| 28 | CORO1C | 0,9466580 | -1,1415627 | -0,2093519 | 0,0669668 | 1,2839478 |
| 29 | CRLF1 | 0,8737347 | -1,2295840 | -0,2532929 | 0,3386334 | 1,1442435 |
| 30 | DEPDC1 | 0,9845339 | -0,6106532 | -0,4675510 | -0,4167317 | 1,4949359 |
| 31 | DKK1 | 0,8821380 | -0,9817625 | 0,4611644 | -0,6583844 | 1,1789825 |
| 32 | DLGAP5 | 0,9526192 | -0,4071236 | -0,5167114 | -0,5726172 | 1,4964522 |
| 33 | DLX2 | 0,9919620 | -0,9170141 | -0,0737547 | -0,4164363 | 1,4072051 |
| 34 | FANCD2 | 0,9439479 | -0,3674607 | -0,5355047 | -0,5902787 | 1,4932442 |
| 35 | FBN2 | 0,9077145 | -0,4919423 | 0,0647766 | -0,9402286 | 1,3673942 |
| 36 | FGF5 | 0,9668935 | -0,4803065 | -0,4803065 | -0,5388162 | 1,4994293 |
| 37 | FLJ21369 | 0,9431176 | -1,1937667 | 0,0416944 | -0,0983329 | 1,2504052 |
| 38 | FOXC2 | 0,8900783 | -1,2921136 | -0,0141337 | 0,1647070 | 1,1415403 |
| 39 | FOXL1 | 0,9764047 | -1,0727964 | -0,1366139 | -0,1366139 | 1,3460242 |
| 40 | FOXM1 | 0,8974514 | -0,2339765 | -0,3263069 | -0,8778350 | 1,4381184 |

| | | | | | | |
|---|---|---|---|---|---|---|
| 41 | GGT2 | 0,8779367 | -1,3062925 | -0,0187763 | 0,2068242 | 1,1182446 |
| 42 | GLIPR1 | 0,8970130 | -1,2877341 | 0,0178723 | 0,1176560 | 1,1522059 |
| 43 | GSTA1 | 0,9984666 | -0,8926778 | -0,2069956 | -0,3321039 | 1,4317773 |
| 44 | HERC4 | 0,9975610 | -0,7865028 | -0,3632989 | -0,3162796 | 1,4660813 |
| 45 | IQGAP3 | 0,9480837 | -0,3950796 | -0,3950796 | -0,6947939 | 1,4849531 |
| 46 | KIAA0101 | 0,9719012 | -1,0938888 | -0,1191994 | -0,1191994 | 1,3322876 |
| 47 | KIAA1524 | 0,9470137 | -0,4004145 | -0,3543214 | -0,7247463 | 1,4794821 |
| 48 | KIF11 | 0,9407793 | -0,3554806 | -0,4524612 | -0,6782785 | 1,4862203 |
| 49 | KIF14 | 0,9695554 | -0,5000000 | -0,5000000 | -0,5000000 | 1,5000000 |
| 50 | KIF18A | 0,9453488 | -0,4314037 | -0,2491982 | -0,7822367 | 1,4628386 |
| 51 | KIF20A | 0,9192348 | -0,3115347 | -0,3075881 | -0,8342653 | 1,4533881 |
| 52 | KRT19 | 0,8498145 | -1,0080465 | -0,6215852 | 0,4476482 | 1,1819836 |
| 53 | LINC00152 | 0,9531490 | -1,1456947 | -0,1507769 | 0,0062736 | 1,2901979 |
| 54 | LMNB1 | 0,9100954 | -0,2490742 | -0,4163549 | -0,7949407 | 1,4603698 |
| 55 | LMO7DN | 0,8631226 | -1,3443767 | 0,1928308 | 0,0795680 | 1,0719779 |
| 56 | LPXN | 0,9435876 | -1,1918809 | -0,0320262 | -0,0320262 | 1,2559333 |
| 57 | LY6K | 0,9989424 | -0,8064829 | -0,3273799 | -0,3273799 | 1,4612427 |
| 58 | MIR197 | 0,8601863 | -0,5589223 | -0,9422120 | 0,1704206 | 1,3307138 |
| 59 | MKI67 | 0,9572034 | -0,4302665 | -0,4302665 | -0,6326265 | 1,4931595 |
| 60 | MYBL1 | 0,9947402 | -0,7064580 | -0,3882869 | -0,3882869 | 1,4830319 |
| 61 | MYPN | 0,9753562 | -0,5789238 | -0,5537971 | -0,3600918 | 1,4928127 |
| 62 | NCAPG | 0,9335668 | -0,3221996 | -0,5059816 | -0,6576435 | 1,4858247 |
| 63 | NETO2 | 0,9804027 | -0,5864869 | -0,4998585 | -0,4097443 | 1,4960897 |
| 64 | NGF | 0,9127119 | -1,1415165 | -0,3353733 | 0,2415228 | 1,2353670 |
| 65 | NUSAP1 | 0,9076644 | -0,2824041 | -0,2824041 | -0,8753938 | 1,4402019 |
| 66 | PEAR1 | 0,9520433 | -1,1194332 | -0,2336012 | 0,0532400 | 1,2997943 |
| 67 | PEG10 | 0,9562570 | -0,4262600 | -0,4262600 | -0,6398567 | 1,4923767 |
| 68 | PLK1 | 0,9422939 | -0,3756534 | -0,3756534 | -0,7278724 | 1,4791791 |
| 69 | PRC1 | 0,9695554 | -0,5000000 | -0,5000000 | -0,5000000 | 1,5000000 |
| 70 | PRR11 | 0,9670243 | -0,5829566 | -0,1490193 | -0,7226030 | 1,4545789 |
| 71 | PRRX2 | 0,9313195 | -0,9983736 | 0,2942949 | -0,5614094 | 1,2654881 |
| 72 | PRUNE2 | 0,8660440 | -1,1516991 | -0,4088930 | 0,4003804 | 1,1602117 |
| 73 | SCFD2 | 0,9440573 | -0,7922106 | 0,1762234 | -0,7292208 | 1,3452080 |
| 74 | SERTAD2 | 0,9663666 | -1,0775678 | -0,2410438 | -0,0157413 | 1,3343529 |
| 75 | SH2D4A | 0,9435067 | -1,1476509 | -0,2112938 | 0,0816066 | 1,2773381 |
| 76 | SHCBP1 | 0,9606906 | -0,4473652 | -0,4283044 | -0,6188158 | 1,4944854 |
| 77 | SLC17A9 | 0,9107927 | -0,9576815 | -0,5886196 | 0,2531266 | 1,2931744 |
| 78 | SLC7A1 | 0,8866955 | -1,3073067 | 0,1891266 | -0,0021661 | 1,1203462 |
| 79 | SMS | 0,9861292 | -1,0165347 | -0,1811246 | -0,1811246 | 1,3787839 |
| 80 | SMYD2 | 0,9767701 | -1,0737696 | -0,1147977 | -0,1565474 | 1,3451146 |
| 81 | SND1-IT1 | 0,9911941 | -0,6630613 | -0,4132507 | -0,4132507 | 1,4895628 |
| 82 | SPAG5 | 0,9747816 | -0,5301932 | -0,4760453 | -0,4933792 | 1,4996176 |
| 83 | TK1 | 0,9194475 | -0,2882781 | -0,3854581 | -0,7903757 | 1,4641120 |

| | | | | | | |
|---|---|---|---|---|---|---|
| 84 | TM4SF1 | 0,9029901 | -1,2208552 | 0,3103213 | -0,2513720 | 1,1619059 |
| 85 | TMEM14A | 0,9486901 | -1,1620589 | -0,1309735 | 0,0154462 | 1,2775862 |
| 86 | TMSB10 | 0,9584464 | -1,0807462 | 0,1383278 | -0,3560686 | 1,2984869 |
| 87 | TNFRSF12A | 0,8907786 | -1,2518115 | -0,1556301 | 0,2463910 | 1,1610506 |
| 88 | TOP2A | 0,8382911 | -0,0102496 | -0,6744114 | -0,7325379 | 1,4171988 |
| 89 | TPX2 | 0,9373593 | -0,3630396 | -0,3507059 | -0,7590330 | 1,4727785 |
| 90 | TRAJ46 | 0,9486506 | -1,1791821 | 0,0089647 | -0,0948710 | 1,2650884 |
| 91 | TRIB1 | 0,9223123 | -0,6191533 | -0,7940886 | 0,0039666 | 1,4092753 |
| 92 | UCHL1 | 0,9694079 | -1,1046476 | -0,1101503 | -0,1101503 | 1,3249481 |
| 93 | VAT1L | 0,9501265 | -0,4304336 | -0,6323241 | -0,4304336 | 1,4931912 |
| 94 | XYLT1 | 0,9844019 | -0,5938379 | -0,4170548 | -0,4851267 | 1,4960194 |
| 95 | ZNF185 | 0,8901705 | -1,2964101 | 0,2154171 | -0,0462928 | 1,1272858 |

| Cluster 6 | | | | | | |
|---|---|---|---|---|---|---|
| | | | **value** | | | |
| | **gene** | **score** | **C0** | **C1** | **C2** | **C3** |
| 1 | ABCA6 | 0,9525048 | 1,2877365 | 0,0995050 | -0,2637288 | -1,1235126 |
| 2 | ABCA8 | 0,9159840 | 0,9224397 | -0,4968921 | 0,7389766 | -1,1645243 |
| 3 | ABCA9 | 0,9896959 | 1,2412941 | -0,0167792 | -0,0167792 | -1,2077358 |
| 4 | ACVR2A | 0,9062180 | 0,7009772 | 0,1424725 | 0,6109002 | -1,4543499 |
| 5 | ADM | 0,9884582 | 1,0450597 | -0,0444796 | 0,3379496 | -1,3385297 |
| 6 | ALDH3A2 | 0,9143351 | 1,3779140 | 0,0098203 | -0,4308524 | -0,9568819 |
| 7 | ANGPTL2 | 0,9690933 | 1,3609694 | -0,1448893 | -0,1679583 | -1,0481218 |
| 8 | APOD | 0,9380238 | 1,4292203 | -0,2617601 | -0,2617601 | -0,9057000 |
| 9 | ARHGAP12 | 0,9850948 | 1,2193329 | 0,0463200 | -0,0369477 | -1,2287053 |
| 10 | BMPER | 0,8131799 | 1,2640964 | -0,9819644 | 0,3050322 | -0,5871642 |
| 11 | BRINP1 | 0,9043215 | 1,3800257 | 0,0226435 | -0,4654576 | -0,9372116 |
| 12 | C1R | 0,9590991 | 1,3998555 | -0,2586806 | -0,1691220 | -0,9720528 |
| 13 | C1S | 0,9913673 | 1,1227542 | -0,2576852 | 0,3733343 | -1,2384033 |
| 14 | CACNG8 | 0,9982975 | 1,2285173 | -0,2351807 | 0,1896539 | -1,1829905 |
| 15 | CELF2 | 0,8677785 | 1,3088190 | -0,8567105 | 0,2487293 | -0,7008378 |
| 16 | CTNS | 0,9870240 | 1,1291749 | 0,0890745 | 0,0890745 | -1,3073238 |
| 17 | CYBRD1 | 0,9889356 | 1,2548100 | -0,0308421 | -0,0308421 | -1,1931259 |
| 18 | DKK2 | 0,9731872 | 0,9565586 | -0,0352908 | 0,4502402 | -1,3715079 |
| 19 | DPP4 | 0,9445840 | 1,3507519 | -0,5936922 | 0,1393646 | -0,8964242 |
| 20 | DPT | 0,9960547 | 1,1117063 | -0,1143598 | 0,2930594 | -1,2904058 |
| 21 | DRAM1 | 0,9798302 | 1,3497737 | -0,3205264 | 0,0077262 | -1,0369735 |
| 22 | FAM20A | 0,9813359 | 1,1424956 | -0,3836771 | 0,4141879 | -1,1730064 |
| 23 | FBLN1 | 0,9853542 | 1,3288833 | -0,3053091 | 0,0436422 | -1,0672164 |
| 24 | FIBIN | 0,8627578 | 0,6030540 | -0,0742172 | 0,8515976 | -1,3804344 |
| 25 | FMOD | 0,8476691 | 0,7947125 | -0,6162079 | 0,9039214 | -1,0824260 |
| 26 | FOS | 0,8355584 | 1,0100942 | 0,6091982 | -0,4239434 | -1,1953490 |
| 27 | GAA | 0,9412241 | 0,9532072 | -0,4012214 | 0,6668945 | -1,2188803 |
| 28 | GAS1 | 0,9476324 | 0,8914885 | -0,2136030 | 0,6433652 | -1,3212506 |
| 29 | HERC2 | 0,9933866 | 1,2330330 | -0,0420358 | 0,0244311 | -1,2154283 |
| 30 | HEXB | 0,9453098 | 1,3249086 | 0,0540240 | -0,3080406 | -1,0708920 |
| 31 | HSPA4L | 0,9782800 | 1,2512164 | 0,0479806 | -0,1072891 | -1,1919079 |
| 32 | ICAM1 | 0,8749482 | 0,9058619 | -0,6565103 | 0,8019634 | -1,0513150 |
| 33 | IL1R1 | 0,9600606 | 1,3952151 | -0,4005875 | -0,0510726 | -0,9435550 |
| 34 | IL6ST | 0,9592794 | 1,3939849 | -0,2165530 | -0,1907600 | -0,9866720 |
| 35 | KIT | 0,8710060 | 1,3718210 | -0,8068314 | 0,1096298 | -0,6746194 |
| 36 | LCE1C | 0,9519950 | 0,9148712 | 0,2554021 | 0,2554021 | -1,4256754 |
| 37 | LSAMP | 0,9492336 | 1,3745997 | -0,0794910 | -0,2832033 | -1,0119055 |
| 38 | MDM2 | 0,8506981 | 0,7213076 | 0,6333259 | 0,0846208 | -1,4392543 |
| 39 | MFAP4 | 0,9898472 | 1,3063604 | -0,1769960 | -0,0059501 | -1,1234142 |
| 40 | MME | 0,9995382 | 1,2227703 | -0,1683642 | 0,1512511 | -1,2056572 |

| | | | | | | |
|---|---|---|---|---|---|---|
| 41 | MTSS1L | 0,9833271 | 1,0492506 | -0,2188139 | 0,4526472 | -1,2830839 |
| 42 | MYO1D | 0,9793475 | 1,3098991 | -0,4234904 | 0,1534363 | -1,0398450 |
| 43 | NFKBIZ | 0,9092357 | 1,2954594 | 0,1875033 | -0,4356725 | -1,0472903 |
| 44 | PAPPA | 0,8661122 | 0,5982307 | 0,3451216 | 0,5476507 | -1,4910030 |
| 45 | PCDHB7 | 0,9379033 | 1,3280634 | 0,0683858 | -0,3400795 | -1,0563697 |
| 46 | PCSK7 | 0,8020695 | 0,9845832 | 0,6682434 | -0,4961643 | -1,1566623 |
| 47 | PDGFD | 0,9827921 | 1,2987689 | -0,4035104 | 0,1645940 | -1,0598525 |
| 48 | PDPN | 0,9911794 | 1,2839574 | -0,1167402 | -0,0106221 | -1,1565951 |
| 49 | PLXNC1 | 0,9355737 | 1,4199430 | -0,1900301 | -0,3056771 | -0,9242358 |
| 50 | PTGDS | 0,9956936 | 1,2357970 | -0,2883221 | 0,2121277 | -1,1596025 |
| 51 | RAB29 | 0,9623336 | 1,2739995 | 0,0871438 | -0,2099466 | -1,1511966 |
| 52 | RCAN2 | 0,9233436 | 1,4325139 | -0,5388687 | -0,0875014 | -0,8061438 |
| 53 | SECTM1 | 0,9673543 | 1,1477701 | 0,2022116 | -0,0706280 | -1,2793537 |
| 54 | SERPINF1 | 0,9532127 | 1,4062187 | -0,2347313 | -0,2107378 | -0,9607496 |
| 55 | SERPING1 | 0,9639445 | 1,2599811 | -0,5433355 | 0,2975902 | -1,0142357 |
| 56 | SFRP2 | 0,8262352 | 0,4879805 | 0,0708254 | 0,8609319 | -1,4197377 |
| 57 | SLC1A3 | 0,9271886 | 1,4108317 | -0,1194300 | -0,3615958 | -0,9298058 |
| 58 | SLC40A1 | 0,9896370 | 1,2425482 | -0,0180700 | -0,0180700 | -1,2064083 |
| 59 | SMPDL3A | 0,9833706 | 1,0644665 | 0,1086932 | 0,1772739 | -1,3504336 |
| 60 | SNED1 | 0,9850567 | 1,2728858 | -0,0303394 | -0,0704192 | -1,1721273 |
| 61 | SNORA11 | 0,8388690 | 0,5260639 | 0,0183091 | 0,8624391 | -1,4068121 |
| 62 | SNORA72 | 0,8270676 | 0,5365734 | 0,5365734 | 0,4247684 | -1,4979152 |
| 63 | SNORD116-2 | 0,8198534 | 0,5000000 | 0,5000000 | 0,5000000 | -1,5000000 |
| 64 | SNX2 | 0,9594109 | 1,2007664 | 0,1906013 | -0,1694333 | -1,2219344 |
| 65 | SULF2 | 0,8183675 | 0,4594405 | 0,2880002 | 0,7277870 | -1,4752276 |
| 66 | THBS2 | 0,8591600 | 0,7628941 | -0,5158489 | 0,9063747 | -1,1534199 |
| 67 | TMTC1 | 0,9837387 | 1,0957131 | 0,1176731 | 0,1176731 | -1,3310593 |
| 68 | TNXB | 0,9841853 | 1,2997272 | -0,0802523 | -0,0802523 | -1,1392226 |
| 69 | TP53INP1 | 0,9052519 | 0,8235911 | 0,4674565 | 0,1512761 | -1,4423237 |
| 70 | TPP1 | 0,8570559 | 1,2625075 | 0,3054472 | -0,5796053 | -0,9883494 |
| 71 | WLS | 0,9902671 | 1,2244861 | 0,0002587 | 0,0002587 | -1,2250035 |
| 72 | ZMYM6NB | 0,9226857 | 0,7575268 | 0,0286326 | 0,6355870 | -1,4217463 |
| 73 | ZNF600 | 0,9743478 | 1,3183860 | -0,4550603 | 0,1523376 | -1,0156632 |
| 74 | ZNF608 | 0,9359521 | 1,4009479 | -0,1140303 | -0,3301986 | -0,9567189 |
| 75 | ZNF836 | 0,9800364 | 1,3519960 | -0,2770995 | -0,0287139 | -1,0461827 |
